# A highly immunogenic and effective measles virus-based Th1-biased COVID-19 vaccine

**DOI:** 10.1073/pnas.2014468117

**Published:** 2020-11-30

**Authors:** Cindy Hörner, Christoph Schürmann, Arne Auste, Aileen Ebenig, Samada Muraleedharan, Kenneth H. Dinnon, Tatjana Scholz, Maike Herrmann, Barbara S. Schnierle, Ralph S. Baric, Michael D. Mühlebach

**Affiliations:** ^a^Product Testing of Immunological Medicinal Products for Veterinary Use, Division of Veterinary Medicine, Paul-Ehrlich-Institut, D-63225 Langen, Germany;; ^b^German Center for Infection Research, D-63225 Langen, Germany;; ^c^Department of Microbiology & Immunology, University of North Carolina at Chapel Hill, Chapel Hill, NC 27599;; ^d^Division of Virology, Paul-Ehrlich-Institut, D-63225 Langen, Germany;; ^e^Pathogenesis of Respiratory Viruses, Division of Veterinary Medicine, Paul-Ehrlich-Institut, D-63225 Langen, Germany;; ^f^Department of Epidemiology, University of North Carolina at Chapel Hill, Chapel Hill, NC 27599;; ^g^Rapidly Emerging Antiviral Drug Discovery Initiative, University of North Carolina at Chapel Hill, Chapel Hill, NC 27599

**Keywords:** SARS-CoV-2, COVID-19, measles vaccine platform, effective immunity, Th1 immune bias

## Abstract

The COVID-19 pandemic is caused by severe acute respiratory syndrome coronavirus-2 (SARS-CoV-2) and has spread worldwide, with millions of cases and more than 1 million deaths to date. The gravity of the situation mandates accelerated efforts to identify safe and effective vaccines. Here, we generated measles virus (MeV)-based vaccine candidates expressing the SARS-CoV-2 spike glycoprotein (S). Insertion of the full-length S protein gene in two different MeV genomic positions resulted in modulated S protein expression. The variant with lower S protein expression levels was genetically stable and induced high levels of effective Th1-biased antibody and T cell responses in mice after two immunizations. In addition to neutralizing IgG antibody responses in a protective range, multifunctional CD8^+^ and CD4^+^ T cell responses with S protein-specific killing activity were detected. Upon challenge using a mouse-adapted SARS-CoV-2, virus loads in vaccinated mice were significantly lower, while vaccinated Syrian hamsters revealed protection in a harsh challenge setup using an early-passage human patient isolate. These results are highly encouraging and support further development of MeV-based COVID-19 vaccines.

## Significance

The COVID-19 pandemic has already caused over 1 million deaths. Therefore, effective vaccine concepts are urgently needed. In search of such a concept, we have analyzed a measles virus-based vaccine candidate targeting SARS-CoV-2. Using this well-known, safe vaccine backbone, we demonstrate here induction of functional immune responses in both arms of adaptive immunity, yielding antiviral efficacy in vivo with the desired immune bias. Consequently, no immunopathologies became evident during challenge experiments. Moreover, the candidate still induces immunity against the measles, recognized as a looming second menace, when countries are forced to stop routine vaccination campaigns in the face of COVID-19. Thus, a bivalent measles-based COVID-19 vaccine could be the solution for two significant public health threats.

Severe acute respiratory syndrome coronavirus-2 (SARS-CoV-2) belongs to *Coronaviridae* family and emerged toward the end of 2019 as causative agent of pneumonia in the Hubei province in China (1). The World Health Organization named the disease Corona Virus Disease-2019 (COVID-19), and officially declared the pandemic state on March 11, 2020. Human coronaviruses have been known for decades as one of the causative agents of the common cold, but two previous coronavirus outbreaks, caused by the severe acute respiratory syndrome virus (SARS-CoV-1) and the Middle East respiratory syndrome virus (MERS-CoV), have demonstrated the remarkable pathogenic potential of human beta coronaviruses. Around 10,000 people have been infected by SARS and MERS, which has resulted in a death toll of about 1,500 patients, but the outbreaks remained largely confined in terms of time or spread, respectively. In contrast, SARS-CoV-2 spreads effectively and at a rapid pace by direct transmission, with a reproductive number R_0_ of at least 2 to 2.5 (2, 3). Due to high transmissibility and extensive community spread, this novel coronavirus has already caused over 36.2 million infections and over 1 million deaths (as of October 9, 2020; https://www.who.int/emergencies/diseases/novel-coronavirus-2019), while worldwide shutdowns of social life and economy to confine the spread of this respiratory virus have considerable impacts.

After the emergence of SARS in 2002 and then MERS in 2012, vaccine development efforts have been initiated, including the use of recombinant measles virus (MeV) vaccine as a platform concept (4), to develop vector vaccine candidates against both agents, and showed promising results. Recombinant MeV vectors encoding the unmodified SARS-CoV Spike protein induced high titers of neutralizing antibodies as well as IFN-γ T cell responses (5, 6) and conferred protection to immunized animals upon pathogen challenge by lowering virus titers more than 100-fold (5). For MERS, we have demonstrated that high titers of neutralizing antibodies as well as effective and polyfunctional T cell responses were induced in vaccinated animals (7, 8) and conferred protection (7). Based on these data, an MeV-based MERS vaccine candidate has been selected by the Coalition for Epidemic Preparedness Initiative for further clinical development (http://www.cepi.net/research_dev/our-portfolio).

Here, we explored the potential of recombinant MeV as vectors for the expression of the SARS-CoV-2 spike protein (S) as successfully applied for the development of MERS (7, 8) and SARS (5, 6) vaccine candidates, as well as numerous other pathogens (4). The S glycoprotein was chosen as antigen for its role as primary target of neutralizing antibodies (6, 7) and the exemplary capability of MERS-CoV S protein to trigger strong cell-mediated immune responses when expressed by MeV in our front-runner MERS vaccine candidate (7, 8). The SARS-CoV-2 S protein-encoding gene was inserted into two different positions of the MeV genome to modulate antigen expression, and both recombinant MeVs were successfully rescued. The virus expressing lower S protein levels resulted in stable amplification over at least 10 passages, while impairment of replication was insignificant. Indeed, immunization of IFNAR^−/−^-CD46Ge mice induced strong and functional humoral and cellular immune responses directed against both MeV and SARS-CoV-2 S protein biased for Th1-type T cell and antibody responses. The induced immunity translated in antiviral efficacy in two different challenge models, that is, vaccinated hamsters and mice, thereby illustrating the potential of MeV platform-based COVID-19 vaccine candidates.

## Results

### Generation and Characterization of SARS-CoV-2-S by Recombinant MeV_vac2_.

**Fig. 1.**
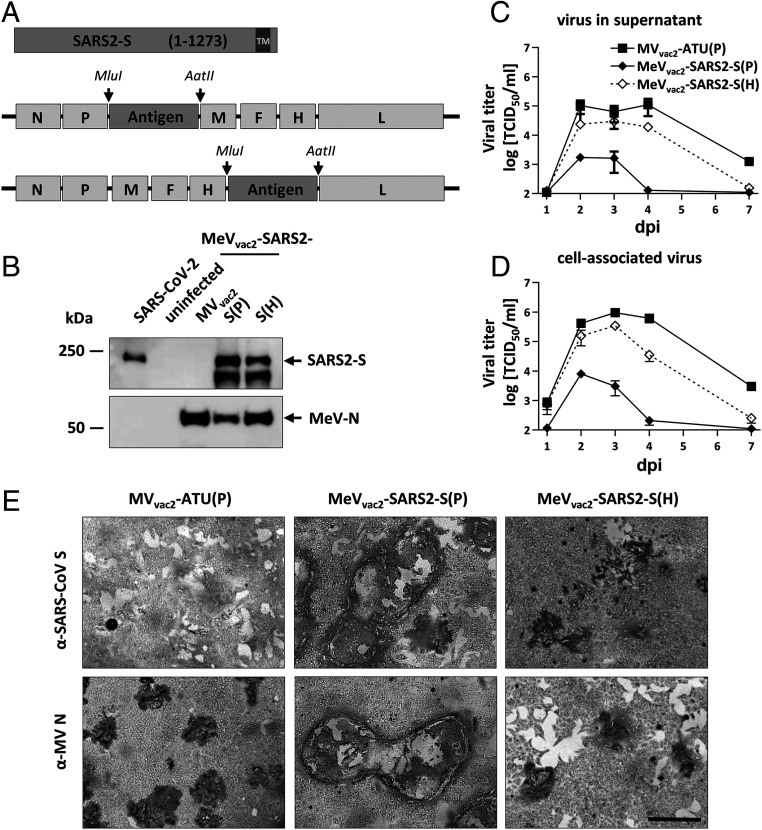
Generation and in vitro characterization of MeV_vac2_-SARS2-S(P) and MeV_vac2_-SARS2-S(H). (*A*) Schematic depiction of full-length SARS-CoV-2 S and recombinant MeV_vac2_ genomes used for expression of this antigen (lower schemes). Antigens or antigen-encoding genes are depicted in dark gray; MeV viral gene cassettes (in light gray) are annotated. *MluI* and *AatII* restriction sites used for cloning of antigen genes into post-P or post-H ATU are highlighted. (*B*) Immunoblot analysis of Vero cells infected at an MOI of 0.01 with MeV_vac2_-SARS2-S(P), MeV_vac2_-SARS2-S(H), or MV_vac2_-ATU(P) (MV_vac2_) as depicted above lanes. Uninfected cells served as mock. Blots were probed using rabbit polyclonal anti-SARS spike antibody (upper blot) or mAb reactive against MeV-N (lower blot). Arrows indicate specific bands. (*C* and *D*) Growth kinetics of recombinant MeV on Vero cells infected at an MOI of 0.03 with MV_vac2_-ATU(P) or MeV_vac2_-SARS2-S encoding extra genes in post H or post P. Titers of samples prepared at indicated time points postinfection (days post infection, dpi) were titrated on Vero cells. Means and SDs of three to five independent experiments are presented. (*E*) SARS-CoV-2 S protein expression in Vero cells was verified via immunoperoxidase monolayer assay; 50× magnification. (Scale bar, 500 μm.)

Since the SARS-CoV and MERS-CoV S proteins have been shown to potently induce humoral and cellular immune responses, the SARS-CoV-2 S protein was chosen as an appropriate antigen to be expressed by the recombinant MeV vaccine platform. A codon-optimized full-length gene encoding SARS-CoV-2 S protein was cloned into two different additional transcription units (ATUs) in the vaccine strain MeV_vac2_ genome, either downstream of the P (post P) or H (post H) gene cassettes (Fig. 1*A*). Recombinant viruses were successfully generated and amplified up to P10 in Vero cells with titers of up to 4 × 10^7^ 50% tissue culture infectious doses (TCID_50_)/mL. The stability of the viral genomes was demonstrated via sequencing after RT-PCR of viruses in P2 or P10. In parallel to Sanger sequencing of the ATU region encompassing the SARS-CoV-2-S gene, the full genome was sequenced using next-generation sequencing (NGS) with a coverage of 4 to 29,683 reads of each position (*SI Appendix*, Fig. S1) in P2. Both methods revealed no mutations across the whole vaccine genomes but a single A to G substitution on position 9 of the noncoding trailer region of the MeV_vac2_-SARS2-S(H) clone used for in vivo studies (GenBank accession no. MW090971).

To verify SARS-CoV-2 S protein expression levels, Western blot analysis of Vero cells infected with the MeV_vac2_-SARS2-S was performed. The S protein expression was slightly attenuated when cells were infected with viruses encoding the antigen in the ATU post-H compared to the post-P constructs (Fig. 1*B*). However, there was less overall viral protein expression in cells infected with post-P construct. Comparative growth kinetics with the vaccine viruses containing the SARS-CoV-2 S gene and the MV_vac2_-ATU(P) control virus revealed that the MeV_vac2_ encoding full-length SARS-CoV-2 S gene in post-P position grew remarkably different than the control virus, with ∼100-fold reduced maximal titers. In contrast, growth of MeV_vac2_-SARS2-S(H) was much closer to MV_vac2_-ATU(P), with only a slight trend for lower titers (Fig. 1 *C* and *D*).

The impaired growth of MeV_vac2_-SARS2-S(P) was accompanied by a hyperfusogenic phenotype (Fig. 1*E* and *SI Appendix*, Fig. S2*A*), which was also observed for the post-H vaccine candidate, but to a lesser extent. Therefore, fusion activity was quantified and compared to the parental MV_vac2_-ATU(P) as well as the MV_NSe_-GFP(N), which is known for its hyperfusogenic phenotype due to a V94M substitution in the F_2_ subunit of the MeV fusion protein (9). MV_vac2_-ATU(P) induced fusion of 16.8 ± 0.8 (mean± SD) Vero cells 30 h after infection. MeV_vac2_-SARS2-S(P) revealed approximately fourfold enhanced fusion activity (syncytia including 70 ± 8 cells), while MeV_vac2_-SARS2-S(H) just fused 41 ± 6 cells, thereby representing an intermediate phenotype. However, fusion activity of the latter was surpassed by MV_NSe_-GFP(N) that fused 56 ± 4 cells in 30 h under the same conditions (*SI Appendix*, Fig. S2*B*).

To investigate whether this increased fusion activity is due to SARS-CoV-2 S protein-mediated cell-to-cell fusion, we expressed the SARS-CoV-2 S protein by transfection of the eukaryotic expression plasmid pcDNA3.1-SARS2-S into SARS-CoV-2 receptor hACE2-negative 293T as well as into receptor-positive Vero cells. Indeed, expression of SARS-CoV-2 S protein induced syncytia of Vero, but not of 293T cells (*SI Appendix*, Fig. S3).

These data demonstrate that the hyperfusogenic phenotype of the SARS-CoV-2 S-encoding MeV is linked to expression of a fusion-active form of the SARS-CoV-2 S protein, indicating that cells infected by the vaccine candidates express a functional S protein. Thus, cloning and rescue of MeVs expressing correctly folded SARS-CoV-2 S was achieved successfully. Since higher S protein expression levels impaired viral replication, MeV_vac2_-SARS2-S(H) was chosen for further characterization in vivo.

### MeV_vac2_-SARS2-S(H) Induces Neutralizing Antibodies against MeV and SARS-CoV-2.

**Fig. 2.**
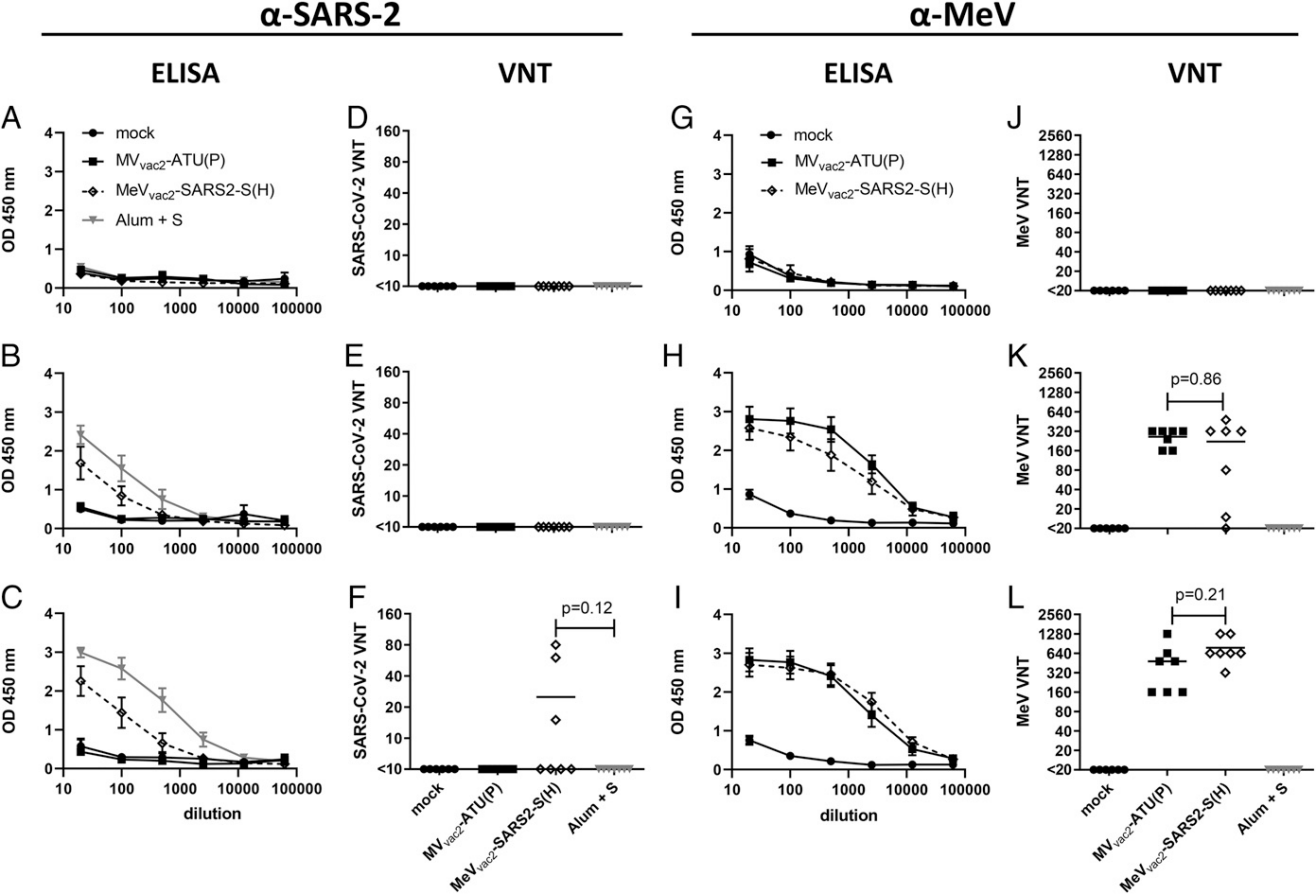
Induction of α-SARS-CoV-2 S and α-MeV specific antibodies. Sera of mice vaccinated on days 0 and 28 with indicated viruses or Alum-adjuvanted S protein were sampled on day 0 (*A*, *D*, *E*, *F*), day 28 after prime immunization (*B*, *E*, *H*, *K*), and day 49 after boost immunization (*C*, *F*, *I*, *L*) and analyzed for antibodies specific for SARS-CoV-2 S or MeV. Medium-inoculated mice served as mock. Pan-IgG binding to recombinant SARS-CoV-2 S (*A*–*C*) or MeV bulk antigens (*G*–*I*) were determined by ELISA via the specific optical density (OD) 450 nm value. Depicted are means and respective SD of the mean (SEM) of each group (*n* = 5 to 7). VNT in vaccinated mice for SARS-CoV-2 (*D*–*F*) or MeV (*J*–*L*) were calculated as reciprocal of the highest dilution abolishing infectivity.

To test the efficacy of MeV_vac2_-SARS2-S(H) in vivo*,* genetically modified IFNAR^−/−^-CD46Ge mice were used, since they are the prime small animal model for analysis of MeV-derived vaccines (10). Groups of six to seven animals were immunized via the intraperitoneal route on days 0 and 28 with 1 × 10^5^ TCID_50_ of MeV_vac2_-SARS2-S(H) or empty MV_vac2_-ATU(P) as a control. As positive control, recombinant SARS-CoV-2 S protein adjuvanted with aluminum hydroxide gel (Alum) was injected subcutaneously, and medium-inoculated mice served as mock controls. Twenty-one days after the second immunization, sera of immunized mice were analyzed in comparison to prebleed and postprime immunization sera, by ELISA on antigen-coated plates, for total IgG antibodies binding to MeV bulk antigens (Fig. 2 *G*–*I*) or SARS-CoV-2 S protein (Fig. 2 *A*–*C*). Sera of mice vaccinated with MeV_vac2_-SARS2-S(H) contained IgG antibodies that bound to SARS-CoV-2 S protein (Fig. 2 *B* and *C*), whereas no antibodies were found in mice before vaccination (Fig. 2*A*), or in MeV or mock-immunized control mice. Moreover, final sera of mice vaccinated with any recombinant MeV had IgG in the serum binding to MeV bulk antigens, indicating at least one successful vaccination with MeVs and general vector immunogenicity (Fig. 2 *G*–*I*). The control S protein vaccine did induce higher levels of S protein-binding IgG than MeV_vac2_-SARS2-S(H) (Fig. 2*C*).

We next determined the neutralizing antibody responses against SARS-CoV-2 (Fig. 2 *D*–*F*) or MeV (Fig. 2 *J*–*L*). Most mice immunized with recombinant MeV, including those receiving the control virus, had developed MeV neutralizing antibody titers (virus neutralization titer [VNT]) after the first immunization (Fig. 2*K*). However, two mice of the MeV_vac2_-SARS2-S(H) cohort initially reacted only weakly, and another mouse reacted not at all, reflecting individual differences in response to immunization. All animals had developed neutralizing antibodies after the second immunization, and a threefold increase was observed upon the second immunization (220 to 762 VNT; Fig. 2 *K* and *L*). Neutralizing antibodies against SARS-CoV-2 were detected in mice vaccinated with MeV_vac2_-SARS2-S(H) after the second immunization, and reached a titer of 15 to 80 in three out of seven mice (Fig. 2*F*). These titers were in the range of human reconvalescent sera tested in parallel (VNT 10 to 60; *SI Appendix*, Fig. S4). No VNTs against MeV or SARS-CoV-2 were detected in control mice inoculated with medium alone. Interestingly, the Alum-adjuvanted recombinant S protein did not induce any neutralizing antibodies despite higher binding IgG levels in ELISA, indicating that these antibodies bind to other epitopes of S or with lower affinity than those induced by the MeV-based vaccine candidate. In summary, the SARS-CoV-2 S protein-expressing MeV elicited robust neutralizing antibody responses against MeV and SARS-CoV-2.

### Splenocytes of Animals Vaccinated with MeV_vac2_-SARS2-S(H) React to SARS-CoV-2 S Protein-Specific Stimulation.

To assess the ability of MeV_vac2_-SARS2-S(H) to induce SARS-CoV-2−specific cellular immune responses, splenocytes of vaccinated animals were analyzed for antigen-specific IFN-γ secretion by Enzyme-Linked Immunosorbent Spot (ELISpot) assay. Toward this, antigen-specific T cells were restimulated by cocultivation with the syngeneic murine dendritic cell (DC) cell lines JAWSII or DC2.4 stably expressing the SARS-CoV-2 S protein. For JAWSII cells, bulk cultures of transduced cells were obtained by flow cytometric sorting. For DC2.4 cells, single-cell clones were generated by limiting dilution of sorted bulk cultures. Antigen expression by transduced DCs was verified by Western blot analysis and flow cytometry (*SI Appendix*, Fig. S5).

**Fig. 3.**
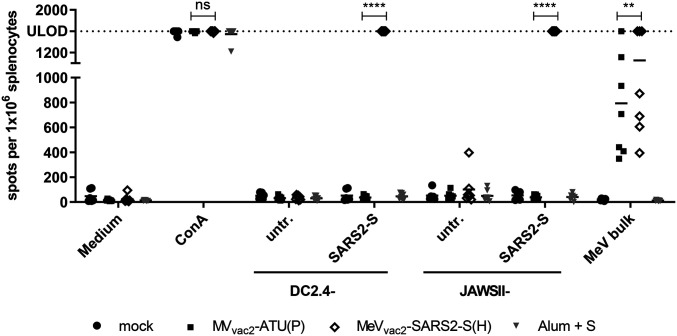
Secretion of IFN-γ after antigen-specific restimulation of splenocytes. IFN-γ ELISpot analysis using splenocytes of mice vaccinated on days 0 and 28 with indicated vaccines, isolated 21 d after boost immunization, and after coculture with DC2.4 or JAWSII dendritic cell lines transgenic for SARS-CoV-2 S (SARS2-S) or untransduced controls (untr.). To analyze cellular responses directed against MeV, splenocytes were stimulated with 10 μg/mL MeV bulk antigens or were left unstimulated as controls (medium). The reactivity of splenocytes was confirmed by ConA treatment (10 μg/mL). The number of cells per 1 × 10^6^ splenocytes represents the amount of cells expressing IFN-γ upon restimulation. Dots represent individual animals; horizontal bars are mean per group (*n* = 6 to 7). Samples above the upper detection limit (ULOD) were displayed as such. For statistical analysis of grouped ELISpot data, two-way ANOVA analysis was applied with paired Tukey’s multicomparison test used as post hoc test; ns, not significant; (*P* > 0.05); **P < 0.01; ****P < 0.0001.

ELISpot assays using splenocytes of vaccinated animals in coculture with DC2.4-SARS2-S cells revealed more than 1,400 IFN-γ secreting cells per 1 × 10^6^ splenocytes after immunization with MeV_vac2_-SARS2-S(H) (Fig. 3). In contrast, coculture with splenocytes of control mice resulted in a background response of less than 50 IFN-γ producing cells per 1 × 10^6^ splenocytes. As expected, restimulation of T cells by DC2.4 presenting no exogenous antigen revealed only reactivity in the range of background (Fig. 3). To rule out clonal or cell line-associated artifacts, antigen-specific IFN-γ secretion by splenocytes of MeV_vac2_-SARS2-S(H)−vaccinated mice was confirmed by stimulation with transgenic JAWSII-SARS2-S bulk cells. These cells also stimulated in excess of 1,400 IFN-γ secreting cells per 1 × 10^6^ splenocytes in animals receiving the recombinant SARS-CoV-2 vaccines, whereas only slight background stimulation was observed by the respective controls. The differences between MeV control and MeV_vac2_-SARS2-S(H) vaccinated mice were statistically significant for both cell lines. Mice vaccinated with Alum-adjuvanted S protein showed no specific reactivity in IFN-γ ELISpot.

Cellular immune responses upon stimulation with MeV bulk antigens were detected in animals that had been vaccinated with any recombinant MeV virus, as expected. While MeV bulk antigens stimulated about 300 to 1,400 IFN-γ secreting cells per 1 × 10^6^ splenocytes of MV_vac2_-ATU(P)−vaccinated animals, splenocytes of MeV_vac2_-SARS2-S(H)−vaccinated animals were in a similar range of 400 to 1,400 IFN-γ secreting cells per 1 × 10^6 ^splenocytes. Splenocytes of all animals revealed a similar basic reactivity to unspecific T cell stimulation, as confirmed by numbers of IFN-γ secreting cells upon concanavalin A (ConA) treatment at the limit of detection. Remarkably, stimulation of splenocytes by DC2.4 expressing SARS-CoV-2-S resulted in at least similar or even higher numbers of IFN-γ^+^ cells than after stimulation by MeV bulk antigens, indicating an extremely robust induction of cellular immunity against this antigen. Taken together, these data show that MeV_vac2_-SARS2-S(H) induces not only humoral but also strong SARS-CoV-2 S protein-specific cellular immune responses.

### SARS-CoV-2 S-Reactive T Cells Are Multifunctional.

To gain more detailed insights into the quality of the observed T cell responses, we further characterized the responsive T cell populations by flow cytometry, determining the expression of IFN-γ, TNF-α, and IL-2 in CD8^+^ and CD4^+^ positive CD3^+^ T cells upon restimulation with SARS-CoV-2 S-presenting DC2.4-SARS2-S cells by intracellular cytokine staining (ICS). As a positive stimulus for T cell activation, ionomycin and phorbol myristate acetate (Ionomycin/PMA) were used. Exocytosis of cytokines was blocked by addition of brefeldin A (10 μg/mL) during stimulation. Cells were permeabilized, labeled, and fixed for flow cytometry. The gating strategy excluded duplicates (*SI Appendix*, Fig. S6, *Middle*, first row), selected for living cells (*SI Appendix*, Fig. S6, *Right*, first row), and separated CD8^+^ and CD4^+^ T cells on CD3^+^ cell populations (*SI Appendix*, Fig. S6, second row). Selected T cells were then analyzed for their expression of IFN-γ, TNF-α, or IL-2, double-positive (*SI Appendix*, Fig. S6, third row), or triple-positive (*SI Appendix*, Fig. S4, fourth row) cells as exemplarily shown for CD4^+^ T cells after restimulation with PMA and ionomycin (*SI Appendix*, Fig. S6).

**Fig. 4.**
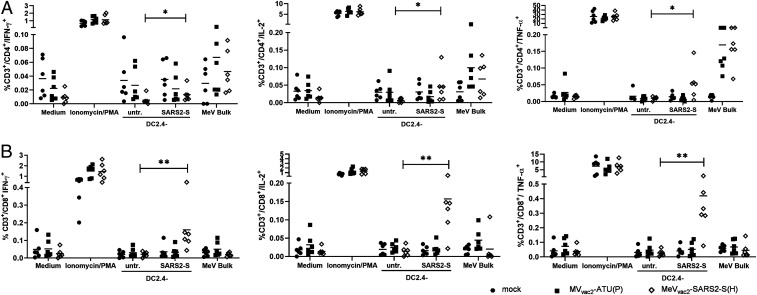
Detection of multifunctional T-cell responses induced by vaccination with MeV_vac2_-SARS2-S(H). Harvested splenocytes of MeV_vac2_-SARS2-S(H)−vaccinated mice (same as depicted in Fig. 3) were restimulated and subjected to intracellular staining (ICS) for IFN-γ (*Left*), TNF-α (*Right*), and IL-2 (*Middle*), and stained for extracellular T-cell markers CD3, CD4, and CD8 for flow cytometry analysis. Quantification of flow cytometry data of (*A*) CD4^+^- and (*B*) CD8^+^-positive T cells after coculture with antigen-presenting DC2.4-SARS2-S or parental DC2.4 control cells, or after incubation with indicated stimuli (MeV bulk antigen [MeV bulk], or untreated cells [mock]); reactivity of splenocytes was confirmed by Ionomycin/PMA treatment (10 μg/mL). Dots represent individual animals; horizontal bars are mean. Mann−Whitney test was used to compare cytokines levels between DC2.4 and DC2.4-SARS2-S restimulated splenocytes in the MeV_vac2_-SARS2-S(H) vaccine group without correction for multiple testing, because of the exploratory character of the study. *P < 0.05; **P < 0.01.

Vaccination with MeV_vac2_-SARS2-S(H) induced a significant amount of SARS-CoV-2 S-specific CD8^+^ T cells expressing either IFN-γ (Fig. 4 *B*, *Left*), IL-2 (Fig. 4 *B*, *Middle*), or TNF-α (Fig. 4 *B*, *Right*), with means between 0.02% and 0.5% of positive cells for each of these cytokines. Among those, a significant fraction of cells proved to be multifunctional, with a mean of 47% of the reactive CD8^+^ cells expressing two cytokines or 13% of responsive CD8^+^ cells being positive for TNF-α, IL-2, and IFN-γ (*SI Appendix*, Fig. S7). A much lower portion of responsive CD4^+^ T cells was observed, varying between 0.01% and 0.07% of CD4^+^ T cells. Among the responsive CD4^+^ cells, 40% expressed two cytokines and 11% were positive for TNF-α, IL-2, and IFN-γ. Moreover, vaccination induced a significant fraction of vector-specific CD4^+^ T cells expressing IFN-γ (Fig. 4 *A*, *Left*), IL-2 (Fig. 4 *A*, *Middle*), or TNF-α (Fig. 4 *A*, *Right*) upon restimulation with MeV bulk antigen. Among those, multifunctional CD4^+^ T cells expressing two or all three cytokines were induced with a mean of about 23% and 6% polyreactive T cells (*SI Appendix*, Fig. S7), respectively. To conclude, vaccination with MeV_vac2_-SARS2-S(H) induces not only IFN-γ, TNF-α, or IL-2 expressing T cells directed against SARS-CoV-2 and MeV but also a significant fraction of multifunctional cytotoxic T cells specific for SARS-CoV-2 S and CD4^+^ T cells specific for MeV antigens, illustrating that a broad and robust SARS-CoV-2−specific immune response is induced by vaccination with MeV_vac2_-SARS2-S(H).

The reactivity of these T cells also became apparent in a proliferative response to antigen-specific stimulation. Using carboxyfluorescein succinimidyl ester (CFSE)-labeled splenocytes and flow cytometry, we detected significant proliferation of both CD4^+^ and CD8^+^ T cells upon stimulation with S-presenting DCs only among the splenocytes of MeV_vac2_-SARS2-S(H)−vaccinated animals (*SI Appendix*, Fig. S8).

### Induced T Cells Reveal Antigen-Specific Cytotoxicity.

**Fig. 5.**
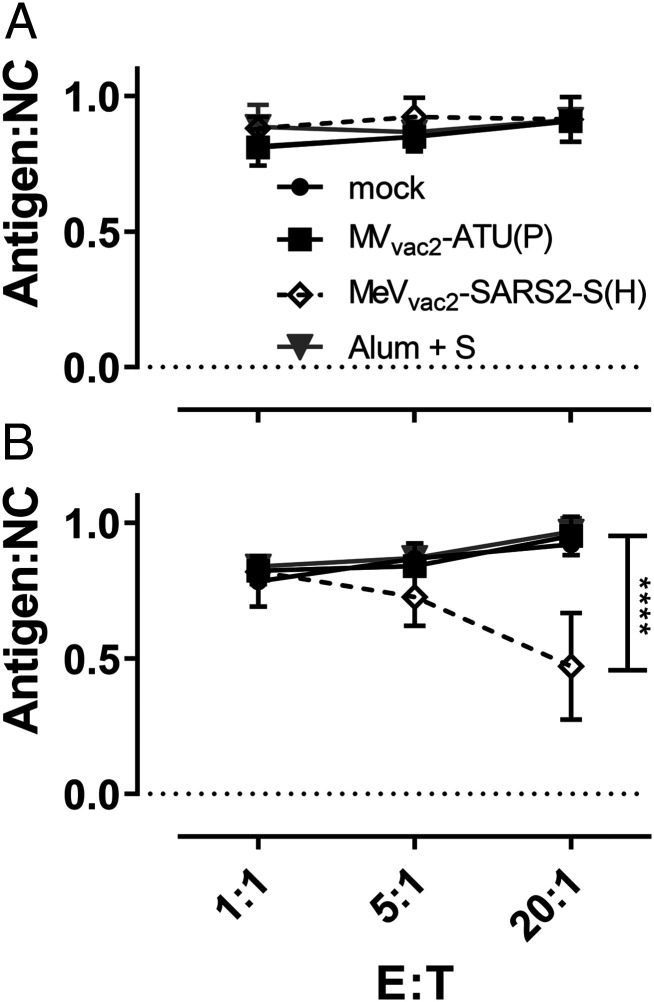
Antigen-specific killing activity of SARS-CoV-2 S-specific T cells. Killing assay using splenocytes of mice vaccinated on days 0 and 28 isolated 21 d after the second immunization. Splenocytes were cocultured (*A*) with DC2.4 or (*B*) with antigen-presenting DC2.4-SARS2-S cells for 6 d. Activated CTLs were then cocultured with EL-4_green_-SARS2-S target cells (Antigen) and EL-4_red_ control cells (NC) at indicated E:T ratios for 4 h. Ratio of living target to nontarget cells (Antigen:NC) was determined by flow cytometry. Depicted are means and SD of each group (open diamonds, MeV_vac2_-SARS2-S(H); filled circles, mock; filled squares, MV_vac2_-ATU(P); gray triangles, S protein + Alum) (*n* = 4 to 6). For statistical analysis, two-way ANOVA analysis was applied with paired Tukey’s multicomparison test used as post hoc test. E:T, effector cell : target cell ratio; ****P < 0.0001.

To demonstrate the effector ability of induced cytotoxic T lymphocytes (CTLs), a killing assay was performed to directly analyze antigen-specific cytotoxicity (Fig. 5). Splenocytes of immunized mice isolated 21 d post boost vaccination were cocultured with DC2.4-SARS2-S or parental DC2.4 cells for 6 d to restimulate antigen-specific T cells. When these restimulated T cells were coincubated with a defined mixture of EL-4_green_-SARS2-S target and EL-4_red_ control cells (ratio ∼1:1), only T cells from MeV_vac2_-SARS2-S(H)−vaccinated mice significantly shifted the ratio of live SARS-CoV-2 S protein-expressing target cells to control cells in a dose-dependent manner (Fig. 5*B*). This antigen-dependent killing was also dependent on restimulation with DC2.4-SARS2-S cells, since unstimulated T cells did not significantly shift the ratios of target to nontarget cells (Fig. 5*A*).

These results indicate that CTLs isolated from MeV_vac2_-SARS2-S(H)−vaccinated mice are capable of lysing cells expressing SARS-CoV-2 S. Neither splenocytes of control mice restimulated with DC2.4-SARS2-S nor splenocytes of SARS-CoV-2 S protein vaccinated mice restimulated with control DC2.4 cells showed such an antigen-specific killing activity, demonstrating that MeV_vac2_-SARS2-S(H) induces fully functional antigen-specific CD8^+^ CTLs.

### Induced Immunity Is Skewed toward Th1-Biased Responses.

**Fig. 6.**
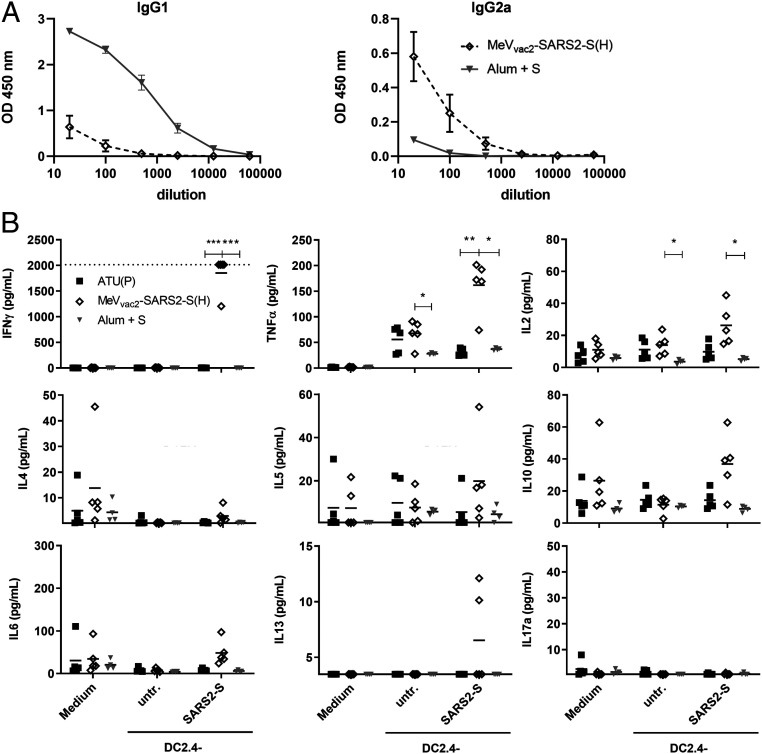
Immune bias of induced responses. To analyze skewing of immune responses toward Th1- or Th2-biased immunity, (*A*) sera and (*B*) splenocytes of vaccinated mice depicted before were analyzed. (*A*) Sera of mice vaccinated on days 0 and 28 with MeV_vac2_-SARS2-S(H) or Alum-adjuvanted S protein already shown in Fig. 2 were analyzed for IgG1-or IgG2a-type antibodies specific for SARS-CoV-2 S. IgG1 (*Left*) or IgG2a (*Right*) binding to recombinant SARS-CoV S were determined by ELISA via the specific optical density (OD) 450-nm value. Depicted are means and respective SD of the mean (SEM) of each group (*n* = 6 to 7). (*B*) Splenocytes of the same mice already shown in Figs. 3–5 and *SI Appendix*, Fig. S8 were analyzed by multiplex cytokine analysis for secretion of typical marker cytokines in the supernatant after restimulation by coculture with antigen-presenting DC2.4-SARS2-S cells. DC2.4 cells served as nonspecific control stimulus. Dots represent individual animals; horizontal bars are mean per group (*n* = 4 to 5). IFN-γ: upper limit of detection (ULOD): 2,015.2 pg/mL; IL-6: ULOD: 3,992.4 pg/mL; IL-17a lower limit of detection (LLOD): 0.473 pg/mL; IL-4 LLOD: 0.095 pg/mL; IL-5 LLOD: 0.685 pg/mL; IL-13 LLOD: 3.463 pg/mL. For statistical analysis of grouped multiplex data, two-way ANOVA analysis was applied with paired Tukey’s multicomparison test as post hoc test. *P < 0.05; **P < 0.01; ***P < 0.001.

While the functionality of both humoral and cellular anti-SARS-CoV-2 immune responses elicited by MeV_vac2_-SARS2-S(H) is reassuring, the SARS-CoV-2 vaccine development has to proceed with some caution because of the potential risk of immunopathogenesis observed in some animal models, such as antibody-dependent enhancement (ADE) and enhanced respiratory disease (ERD) which seem to correlate with a Th2-biased immune response. Since, in mice, IgG1 is a marker for Th2 bias and risk of ADE development, whereas IgG2a antibodies indicate a favorable Th1 bias, IgG subtype-specific ELISA was performed with the sera collected at different time points. Animals vaccinated with Alum-adjuvanted SARS-CoV-2 S protein, a vaccine concept known for its Th2 bias (11, 12), developed high levels of S protein-specific IgG1 antibodies, whereas few S-specific IgG2a antibodies were detected (Fig. 6*A*). In comparison, MeV_vac2_-SARS2-S(H) induced 100-fold fewer IgG1 antibodies, but at least 10-fold higher IgG2a levels (Fig. 6*A*), indicating a favorable Th1 bias in animals immunized with the MeV-derived vaccine candidate.

These findings were confirmed by multiplex cytokine analysis of the cytokine profile in the supernatants of splenocytes from vaccinated animals, which were restimulated using DC2.4 or DC2.4-SARS2-S cells. All splenocytes of representative animals revealed secretion of all cytokines after stimulation with ConA, demonstrating general reactivity of cells and assay (*SI Appendix*, Fig. S9). Most likely due to the low number of S-reactive T cells in animals that had been vaccinated with recombinant SARS-CoV-2 S protein and Alum, no, or minimal, constant cytokine levels were measurable in the supernatants of restimulated splenocytes (Fig. 6*B*). In contrast, splenocytes of animals immunized with MeV_vac2_-SARS2-S(H) reacted specifically with the secretion of IFN-γ, TNF-α, and IL-2 upon restimulation by DC2.4-SARS2-S (Fig. 6*B*), in accordance with ELISpot (Fig. 3) and ICS data (Fig. 4). However, we could observe no or minimal up-regulation of IL-4, IL-5, IL-13, or IL-10, which would have been indicative for a Th2-biased response (Fig. 6*B*). Also, IL-17a or IL-6 indicative of a Th17 or general inflammatory response, respectively, showed minimal changes (Fig. 6*B*).

Thus, both humoral and cellular responses reveal a Th1-biased immunity induced by MeV_vac2_-SARS2-S(H), which indicates a relatively low risk for putatively Th2-mediated immunopathologies.

### MeV-COVID-19 Vaccine-Induced Immune Responses Are Effective In Vivo.

**Fig. 7.**
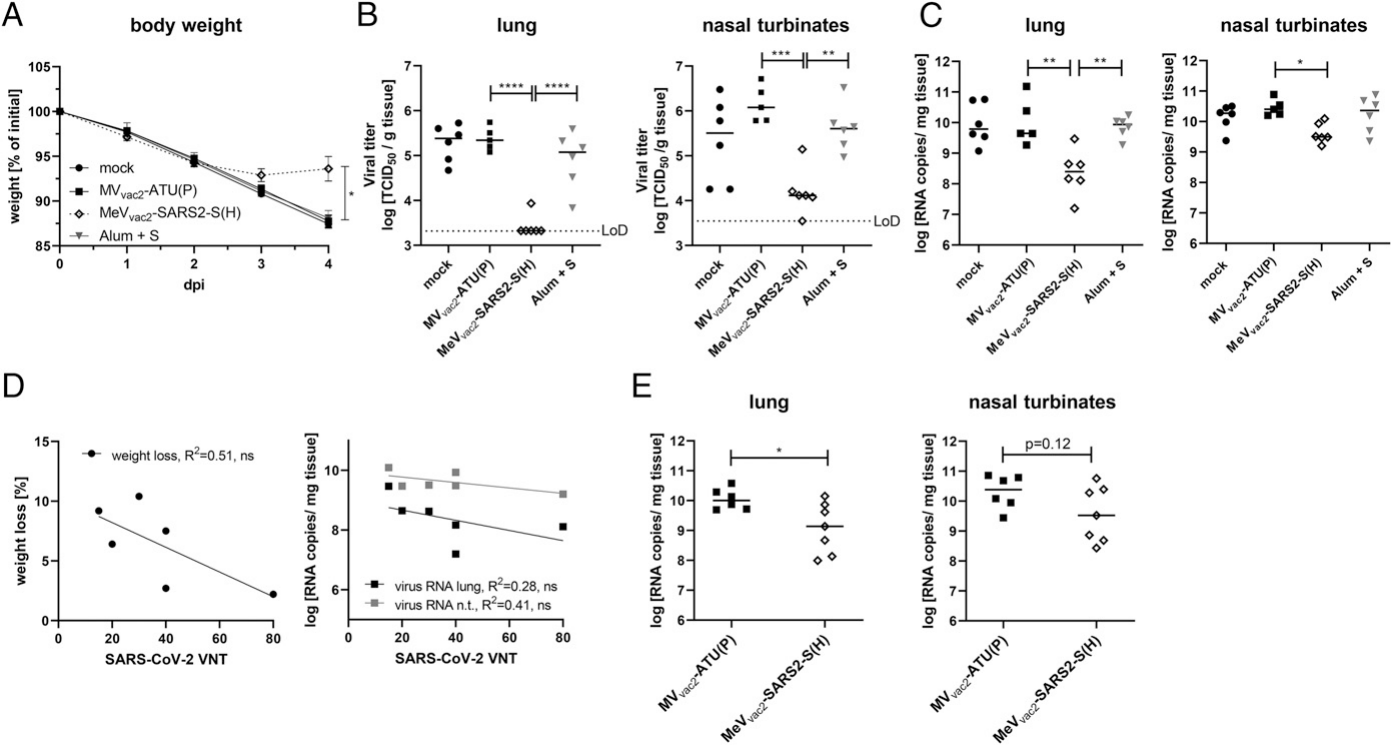
Vaccine efficacy in Syrian hamster and mouse challenge models. Fully immunized Syrian golden hamsters (*A*–*D*) and IFNAR^−/−^-CD46Ge mice (*E*) were intranasally challenged with SARS-CoV-2 or mouse-adapted SARS-CoV-2 MA, respectively, and protection was analyzed by quantifying weight loss (*A*), live virus titration in lungs or nasal turbinates (*B*), or SARS-2 E gene copy numbers via qRT-PCR (*C* and *E*). The limit of detection (LoD) is indicated. (*D*) For the correlation analysis comparing humoral immunity (VNT) of hamsters vaccinated with MeV_vac2_-SARS2-S(H) on days 0 and 21 (sampled on day 35) and challenge outcome, linear regression was performed with the indicated challenge outcome parameters to determine Pearson correlation coefficient (R^2^). For statistical analysis of hamster data, one-way ANOVA was performed in combination with Tukey’s multicomparison test to compare all pair means. For comparison of mouse data between the two groups, unpaired two-tailed *t* test was applied; ns, not significant; (*P* > 0.05); *P < 0.05; **P < 0.01; ***P < 0.001; ****P < 0.0001.

To finally test antiviral efficacy and the protective capacity of the immune responses induced by our COVID-19 vaccine candidate, two different animal models of COVID-19 were used. On the one hand, we established the Syrian golden hamster model for SARS-CoV-2−induced disease, which revealed remarkably severe outcome. Using an available low-passage SARS-CoV-2 patient isolate for intranasal challenge of 6- to 12-wk-old hamsters, the infected animals developed severe pneumonia with an impressive gross pathology (*SI Appendix*, Fig. S10 *A* and *B*) that necessitated killing four out of six animals within 5 d to 6 d postinfection (*SI Appendix*, Fig. S10*C*). To take advantage of this model, hamsters were vaccinated with MeV_vac2_-SARS2-S(H), MV_vac2_-ATU(H) as vector control, Alum-adjuvanted SARS-CoV-2 Spike protein as vaccine control, or medium (mock) in a prime−boost scheme for challenge. These hamsters showed induction of anti-MeV and anti-SARS-CoV-2 humoral immune responses with neutralizing antibody titers against SARS-CoV-2 between 15 and 80 VNT corresponding to 20 to 213 PRNT_50_ (50% plaque reduction neutralization titer), exceeding those of the Alum-S vaccine control group (*SI Appendix*, Fig. S11 *F* and *M*). Upon challenge, the initial weight loss observed in all groups was stopped in the MeV_vac2_-SARS2-S(H) cohort on day 3 postinfection, and animals started to gain weight, whereas all other groups revealed progressive weight loss (Fig. 7*A*). The experiment was stopped 4 d postinfection to determine live virus titers (Fig. 7*B*) as well as virus genome copy numbers (Fig. 7*C*) in relevant tissues, that is, lungs and nasal turbinates. The corresponding data reflected the observations on gross pathology (Fig. 7*A* and *SI Appendix*, Fig. S12): The hamsters vaccinated with MeV_vac2_-SARS2-S(H) had a more than 10-fold reduction in virus genomes in lungs and nasal turbinates than all other groups (Fig. 7*C*), corresponding to absence of live virus in the lungs of five out of six and nasal turbinates of one out of six vaccinated animals (Fig. 7*B*). Thus, vaccination with MeV_vac2_-SARS2-S(H) significantly ameliorated virus loads in infected animals and protected them against severe pathology in this remarkably aggressive challenge model. In this model, SARS-CoV-2 neutralization antibody titers in MeV_vac2_-SARS2-S(H)−vaccinated hamsters showed a tendency to reversely correlate with weight loss and RNA copy numbers in lung and nasal turbinates, indicating some role of neutralizing antibodies in fighting disease progression (Fig. 7*D*).

Our second animal model confirmed these observations. Virus loads of the mouse-adapted SARS-CoV-2 MA (13) were significantly reduced in the lungs of vaccinated mice (corresponding immunity data in *SI Appendix*, Fig. S13), and revealed a tendency for reduced titers in nasal turbinates, as well (Fig. 7*E*), in the absence of gross pathology in both groups. Therefore, both challenge models show protective efficacy of the immune responses induced by MeV_vac2_-SARS2-S(H).

## Discussion

In this study, we aimed to analyze the efficacy of MeV-derived vaccine candidates encoding the Spike glycoprotein S of SARS-CoV-2 to induce functional immune responses to protect against COVID-19. We show that MeV_vac2_-SARS2-S(H) replicated comparably to MeV vaccine strain viruses and was genetically stable over extended passaging. Upon vaccination of mice, it induced robust humoral immune responses of the IgG2a subtype directed against the SARS-CoV-2 spike glycoprotein S with neutralizing activity in a range already shown to be protective by others. In addition, considerable amounts of SARS-CoV-2 S-specific CD4^+^ and CD8^+^ T cells were induced, the major fraction of which were secreting two or even all three cytokines when analyzing for IFN-γ, TNF-α, or IL-2 upon antigen-specific restimulation. These T cells proliferated and specifically depleted antigen-positive target cells in a mixed population. Importantly, all responses were skewed toward Th1-biased immunity. In parallel, the capacity to induce measles-specific immune reactivity remained conserved. Finally, the induced immunity conferred protection in two different animal models of SARS-CoV-2 infection, Syrian golden hamsters inoculated with a low-passage patient isolate and mice challenged with a mouse-adapted recombinant SARS-CoV-2 MA.

This effective MeV Moraten strain-derived recombinant vaccine MeV_vac2_-SARS2-S(H) is a live-attenuated vaccine that encodes the full-length, functional version of the SARS-CoV-2 S protein as the main target for functional antibodies, but also for induction of T cell responses. Vero cells revealed homogenous expression of the SARS-CoV-2 S antigen by Western blot analyses and positive immunostaining of syncytia after infection by MeV_vac2_-SARS2-S(H). Stable antigen expression is a prerequisite for the immune system to encounter the specific antigen to mount robust immune responses and for industrial production of a vaccine. Indeed, IFNAR^−/−^-CD46Ge mice vaccinated with MeV_vac2_-SARS2-S(H) in a prime−boost protocol showed uniform induction of antibodies directed against MeV bulk antigens or SARS-CoV-2 S, which had considerable neutralizing activity against both pathogens. We observe antibody responses in these animals at a level that correlates with protection in mouse challenge models (14), as well as with neutralizing activity we found in the serum of four reconvalescent human patients. These responses were triggered despite the knockout of the type I IFN receptor, which is necessary to allow propagation of MeV in mice (10, 15). This knockout usually should impair the induction of especially humoral immune responses (16). This highlights the remarkable immunogenicity of the MeV vaccine platform technology that also works in this model with partially impaired immune responses.

However, why did not all immunized animals develop neutralizing activity detectable in our assay? Firstly, determination of the VNT relying on 100% pathogen neutralization is obviously a rather harsh assay in the context of SARS-CoV-2, as evidenced by the modest VNT titers published so far, in general, and absence of VNT in the S+Alum vaccinated group despite high amounts of S-binding antibodies. This means that just detectable VNT already indicates considerable neutralizing activity. Indeed, when testing the sera of mice that were later on challenged with SARS-CoV-2 MA in parallel for VNT and PRNT_50_ titers, two animals negative for VNT became positive in PRNT_50_. Secondly, we realized that three of the four animals which did not show a VNT of >10 did not respond well to the prime vaccination, at all. These animals developed no or only a minor VNT against MeV after the first vaccination. This observation is rather unusual, and argues for technical issues during the first vaccination in these animals. Since none of the animals showed VNT against SARS-CoV-2 after one vaccination with the vaccine, it is tempting to speculate that a prime−boost protocol is associated, in this animal model, with maturation of antibodies to generate better neutralizing responses. In hamsters, which fully responded to each vaccination, all six MeV_vac2_-SARS2-S(H)−vaccinated animals developed VNT. On the other hand, all mice, including the three improperly immunized ones, revealed significant, multifunctional T cell responses against SARS-CoV-2 S, which were still recordable 3 wk after the second vaccination, when we already expect constriction of antigen-specific T cell effector populations. These data suggest that anti-S antibody responses mature after repeated vaccination, but, on the other hand, that a one-shot vaccination regime will already induce especially functional memory T cell immune responses, the protective efficacy of which, as well as their duration, has to be demonstrated in future challenge experiments. Moreover, anti-measles immunity is generally stable (17) also after pediatric vaccination (18) and might be a specific advantage of the measles vaccine platform technology, which could transfer also to the additionally encoded antigens.

Also, extended passaging of the vaccine candidate did not result in changes of the vaccine, as revealed by sequencing of the virus after 10 passages starting with a low multiplicity of infection (MOI). This genetic stability indicates that the slight impairment seen in multistep growth curves when compared to a vaccine-strain MeV is not critical for the vaccine’s amplification and therefore crucial for product safety. In accordance with its genetic stability, the minor enhancement of fusion activity can also be regarded as noncritical, especially when considering the fusion activity of MeV used in clinical trials for treatment of tumors. These so-called oncolytic MeV have been used in 15 phase 1 and phase 2 clinical trials, so far. Thereby, advanced-stage tumor patients suffering from different tumor entities have been treated. Despite constituting, in principle, a vulnerable patient collective, application of high doses of nontargeted, fusion-active MeV (up to 1 × 10^11^ TCID_50_) (19) systemically or, for example, directly into the patients’ brains (20) was accompanied by an acceptable safety profile (21). Therefore, the enhancement of fusion activity cannot be expected to be crucial for product safety, while the attenuation of vaccine-strain MeV is multifactorial, anyway, and not just a matter of cell entry tropism and mechanism (22). Likewise, the clinical phase 1 and 2 trials using the MeV vector platform for the generation of bivalent vaccines, which induce immunity against Chikungunya virus (CHIKV) (23, 24), have revealed an extremely beneficial safety profile of this recombinant vaccine concept also in human patients, while signs of efficacy became evident.

In any case, generation of MeV-derived COVID-19 vaccines encoding a less fusion-active variant of the SARS-CoV-2 S glycoprotein might be beneficial to enhance titers of the vaccine virus. In the meantime, stabilized S variants have become available that have attenuated or no cell−cell fusion activity. One variant has a deletion of the multibasic cleavage motif for furin-like proteases at the S1/S2 boundary that facilitates preactivation of S (25). A second variant has proline substitutions at residues 986 and 987, which are stabilizing a prefusion conformation of S (26). Vaccine candidates encoding S with one of these motifs or a combination thereof in a soluble version, as already done for DNA vaccines (27), are under development. These have to show an at least comparable capacity to induce neutralizing antibody responses also in the context of MeV infection, which might be dependent on the respective conformation of the antigen that is expressed by vaccine virus-infected cells in situ.

The induction of the “right” antibodies and T cell responses is especially crucial when taking potential complications into consideration that can be observed when coronavirus encounters “wrong” immune responses that can give rise to immunopathologies after infection. In some infected cats, infection with feline coronavirus causes feline infectious peritonitis, a deadly disease characterized by viral infection of macrophages during the acute phase. Interestingly, the switch of pathology after infection from a rather moderate pathogenesis into an acute, devastating disease can be triggered by vaccination of persistently infected cats and has been attributed to the induction of antibodies that mediate enhancement of the disease, a process called ADE. During COVID-19, ADE might be the cause of the severe cases currently observed. Some case reports indicate that severe disease appeared more frequently in patients with high SARS-CoV-2 IgG levels (28). ADE has been most prominent for dengue virus (DENV) infections, especially in secondary infections with a different DENV serotype where enhancement of disease correlated with the induction of nonneutralizing Abs that can mediate an efficient uptake of the virus into FcR-positive cells such as macrophages and other immune cells (29). Moreover, other immune-related adverse events were described for SARS- and MERS-CoV. When animals were immunized with vaccines that predominantly induce Th2-biased T-helper cell responses, vaccinated mice revealed significantly reduced virus loads after challenge, but also an eosinophilic infiltrate into the lungs accompanied by pathological changes of the lung tissue, so-called ERD (30). Such immunopathologies upon CoV infection are a major concern for diseases pathology and, especially, vaccine development. Thus, Th2-biased immune responses as triggered by alum-adjuvanted whole inactivated virus particles or recombinant proteins should be avoided.

Interestingly, the live-attenuated MeV vaccine is known for a balanced Th1/Th2 bias of induced immune responses, with a bias for Th1 responses at least during the acute phase after vaccination (31). In theory, this should also apply for immune responses induced against all antigens presented during an MeV vaccine virus infection including foreign antigen(s) additionally expressed when MeV is used as vaccine platform. Indeed, our analyses provide evidence that the bias of the immune responses is in favor of Th1 responses, as revealed by the inverted IgG1/IgG2a subtype ratio of antibodies induced against SARS-CoV-2 S by MeV_vac2_-SARS2-S(H) compared to the animals immunized with alum-adjuvanted recombinant S protein. Moreover, the cytokine profile of splenocyte cultures of immunized mice after restimulation of S-specific T cells reveals a respective preferable Th1 bias. Since SARS-CoV-2 and SARS-CoV use the same primary attachment receptor for cell entry, hACE2, and selected hACE2-transgenic mice show differential pathology after inoculation with SARS-CoV-2 (14, 32), studying the impact of the Th1-biased MeV-based immunization in hACE2-transgenic mice during challenge with SARS-CoV-2 will be a matter for future studies. In any case, our challenge data also reveal that MeV-derived COVID-19 vaccines have a low likelihood to trigger immunopathogenesis, but we show considerable antiviral efficacy, especially in the Syrian hamster model. This animal model is susceptible for SARS-CoV-2 infection and suitable for vaccination using recombinant MeV (33); reveals in our setting a moderate to severe, clearly distinguishable pathology; and shows airborne transmissibility from infected to naïve animals. Therefore, this animal model accurately reflects at least some aspects of the course of human disease and reveals the potential of our candidate to vaccinate effectively against COVID-19.

In conclusion, the bivalent MeV/SARS-CoV-2 vaccine candidate has a number of desirable properties with respect to its immunogenicity and efficacy against SARS-CoV-2. Furthermore, the concurrent induction of anti-MeV immunity would allow its use in the context of routine measles immunization schedules. Such an MeV-based COVID-19 vaccine could be included in the currently applied MMR (measles, mumps, rubella) vaccine, providing additional protection against SARS-CoV-2. While there is controversy regarding to what extent it occurs, children do become infected and shed the virus, despite them rarely being severely affected. In any case, preventing or reducing infection or virus shedding from vaccinated children can also help to contain the disease and protect vulnerable patient groups. On the other hand, the vaccinated children themselves could be protected against newly described side effects of COVID-19 infections, such as Multisystem Inflammatory Syndrome, affecting also the cohort of (very) young patients (34). Thereby, the capacity to produce large amounts of vaccine doses as well as the respective distribution network would be available more or less instantly from routine measles vaccine production, but at no impairment of production of other necessary vaccines, since the measles vaccine property is preserved in the proposed vaccine candidate. Especially since vaccination against the measles should not be impaired also during the COVID-19 epidemic, this is a considerable advantage. Otherwise, parallel epidemics with another, even more contagious respiratory virus are looming when vaccination programs against the measles are stopped in favor of COVID-19 vaccination programs. However, while we envision primarily pediatric application for this vaccine candidate, currently available data also suggest applicability of MeV-derived recombinant bivalent vaccines also in vaccinees with preformed anti-measles immunity. The performance of measles-derived recombinant vaccine targeting HIV-1 or CHIKV has been analyzed in vivo in respective models in measles-vaccinated animals. In both mice and nonhuman primates, at least antibodies against the secondary target pathogen were successfully induced also in preimmune animals (35, 36). This was reflected by the outcome of phase I and phase II clinical studies of an MeV-CHIKV vaccine candidate. Also, in human vaccinees, induction of significant anti-CHIKV antibody titers independent from the preimmunity seen in the respective individuals has been observed (23, 24). Future studies have to demonstrate that this is also the case for MeV-derived COVID-19 vaccine candidates. Taken together, MeV_vac2_-SARS2-S(H) is a promising vaccine candidate that warrants further investigation.

## Materials and Methods

Detailed descriptions of the materials, methods, and equipment used in this work, including cells, plasmids, production of lentiviral vectors and generation of antigen-expressing dendritic cell lines, viruses, MeV genome sequence analysis, NGS library preparation and sequencing, RNA sequence analysis, immunoperoxidase monolayer assay, Western blot analysis, animal experiments, total IgG and IgG1-/IgG2a quantification, Th1/Th2 cytokine multiplex assay, virus neutralization test, plaque reduction neutralization test, IFN-γ ELISpot analysis, ICS, T cell proliferation assay, CTL killing assay, virus titers in organs of infected animals, RNA preparation, quantitative RT-PCR, and statistical analyses, are provided in *SI Appendix*, *Supplementary Extended Materials and Methods*.

## Supplementary Material

Supplementary File

Supplementary File

## Data Availability

All data are provided in the manuscript and *SI Appendix*. The sequence data for the vaccine candidate described in this paper have been deposited in the GenBank Data Bank (accession no. MW090971).
