# Supplementary material for: A highly immunogenic and effective measles virus-based Th1-biased COVID-19 vaccine
Source: Proc Natl Acad Sci U S A. 2020 Nov 30;117(51):32657–66. doi: 10.1073/pnas.2014468117 (PMC7768780; doi:10.1073/pnas.2014468117)
Supplement: Supplementary File [file pnas.2014468117.sapp.pdf]

Supplementary Extended Materials and Methods

Cells

Vero (African green monkey kidney) (ATCC# CCL-81), Vero clone E6 (ATCC# CRL-1586), 293T (ATCC CRL-3216) and EL-4 (ATCC TIB-39) cell lines were purchased from ATCC (Manassas, VA, USA) and cultured in Dulbecco's modified Eagle's medium (DMEM, Biowest, Nuaillé, France) supplemented with 10% fetal bovine serum (FBS; Biochrom, Berlin, Germany) and 2 mM L-glutamine (L-Gln; Biochrom). JAWSII mouse dendritic cells (ATCC CRL-11904) were also purchased from ATCC and cultured in MEM-α (GIBCO BRL, Eggenstein, Germany) supplemented with 20% FBS, 2 mM L-Gln, 1 mM sodium pyruvate (Biochrom), and 5 ng/ml murine GM-CSF (Biotechne, Wiesbaden, Germany). DC2.4 mouse dendritic cells (1) were cultured in RPMI containing 10% FBS, 2 mM L-Gln, 1% non-essential amino acids (Biochrom), 10 mM HEPES (pH 7,4), and 50 μM 2-mercaptoethanol (Sigma-Aldrich, Steinheim, Germany). All cells were cultured at 37°C in a humidified atmosphere containing 6% CO_2 for a maximum of 6 months of culture after thawing of the original stock.

Plasmids

The codon-optimized gene encoding full-length SARS-CoV-2 Spike glycoprotein S of isolate Wuhan-Hu-1 (Genebank accession no. MN908947.1) in plasmids pMA-RQ-SARS2-S flanked with *Aat*II/*Mlu*I and *NheI*/*XhoI* restriction sites was obtained by gene synthesis (Invitrogen Life Technology, Regensburg, Germany). The antigen was inserted into plasmids pBRPolIIΔ-MV_{vac2}-GFP(P) or pBRPolIIΔMV_{vac2}-GFP(H) via *Mlu*I/*Aat*II to generate pBRPolII-MV_{vac2}-SARS2-S(P) or pBRPolII-MV_{vac2}-SARS2-S(H). pBRPolIIΔ-MV_{vac2}-GFP(P) or pBRPolIIΔMV_{vac2}-GFP(H) were generated by inserting the immediate early CMV promoter sequence from p(+)PolII-MV_{NSe}-GFP(N) (2), which had been modified by site-directed mutagenesis for deleting the *AatII* restriction sites, into pBR-MV_{vac2}-GFP(P) or pBR-MV_{vac2}-GFP(H) (3). For construction of a lentiviral transfer vector encoding SARS-CoV-2 S directly linked to the *egfp* gene as selection marker, the ORF of SARS-CoV-2 S was inserted via *Nhe*I/*Xho*I into pCSCW2gluc-IRES-GFP (4) to yield pCSCW2-SARS2-S-IRES-GFP. For construction of a eukaryotic expression plasmid encoding SARS-CoV-2 S, the ORF of SARS-CoV-2 S was inserted via *Nhe*I/*Xho*I into pcDNA3.1(+) (Invitrogen Life Technology) to yield pcDNA3.1-SARS2-S.

Production of lentiviral vectors and generation of antigen-expressing dendritic cell lines

Lentiviral vectors were produced and used for the generation of antigen-expressing dendritic cell lines as described before (3). In short, HIV-1-derived particles pseudotyped with VSV-G were generated using a standard three plasmid system, pMD2.G, pCMVΔR8.9 (5) with the transfer vector plasmid pCSCW2-SARS2-S-IRES-GFP in combination with PEI transfection of 293T cells (6). Subsequent purification by filtration and ultracentrifugation of supernatants yielded virus stocks were used to transduce murine DC cell lines, DC2.4 and JAWSII, as well as the murine T cell line EL-4, resulting in DC2.4-SARS2-S, JAWSII-SARS2-S, and EL-4_{green}-SARS2-S, respectively, that express the SARS-CoV-2 S protein and GFP and present the respective peptides via MHC-I. Transduced cultures with 1-10% GFP-positive cells were single cell-sorted (BD FACS Aria™ Fusion) for GFP-expressing cells and subsequently characterized for antigen expression. For JAWSII-SARS2-S, the bulk-sorted cells were used in

www.pnas.org/cgi/doi/10.1073/pnas.2014468117

stimulation experiments. For DC2.4-SARS2-S and EL-4_{green}-SARS2-S, clonal cell lines were generated by limiting dilution of bulk-sorted cells and characterized for marker- and antigen-expression.

Viruses

SARS-CoV-2 S-encoding vaccine candidates MeV_{vac2}-SARS2-S(P) or MeV_{vac2}-SARS2-S(H) were generated as described previously (3, 7). Single syncytia were picked and overlaid onto 50% confluent Vero cells cultured in 6-well plates and harvested as “passage 0” (P0) by scraping and freeze-thaw cycle of cells at the time of maximal infection. Subsequent passages were generated after $TCID_{50}$ titration of infectious virus according to the method of Kaerber and Spaerman (8). Stocks were generated by infection of Vero cells at an MOI = 0.03, and passage 2 (P2) or P3 were used for *in vitro* characterization, while vaccine viruses in P3 or P4 were used for vaccination experiments. Vector control virus MV_{vac2}-ATU(P) (9) was used in P5 for vaccination. SARS-CoV-2 (isolate MUC-IMB1) (kind gift of G. Dobler, Bundeswehr Institute for Microbiology, Germany) (10) was used for SARS-CoV-2 neutralization assays and hamster challenge. It was propagated on Vero E6 cells and was titrated via $TCID_{50}$ as described above for recombinant MeV. All virus stocks were stored in aliquots at -80°C.

Multistep viral growth kinetics were analyzed by infecting Vero cells at an MOI of 0.03 in 96-well plates and incubated at 37ºC. At various time points, supernatants were clarified by centrifugation, and cells were scraped into OptiMEM and subjected to freeze-thaw cycles. Released and cell-associated viral titers were determined by $TCID_{50}$ limited dilution method.

Measles virus genome sequence analysis

The RNA genomes of recombinant MeV in P2 or P10 were isolated from infected Vero cells using the QIAamp Viral RNA Mini Kit (QIAgen, Hilden, Germany) according to the manufacturer’s instructions and resuspended in 50 µL RNase-free water. Viral cDNA was reversely transcribed using Superscript II RT kit (Invitrogen) with 2 µL viral RNA as template and random hexamer primers, according to manufacturer´s instructions. For specific amplification of the SARS-CoV-2 S ORF, the respective genomic regions of recombinant MeV were amplified by PCR using primers binding to sequences flanking the regions of interest and the cDNA as template. Detailed description of primers and procedures are available upon request. The PCR products were directly sequenced (Eurofins Genomics, Ebersberg, Germany).

NGS library preparation and sequencing

Total RNA was isolated from Vero cells after 4 days post infection using the Direct-zol RNA isolation kit (Zymo Research). 1 µg of RNA isolate was subjected to rRNA removal with the NEBNext rRNA Depletion Kit (NEB) using the manufacturer’s recommendations. The whole 10 µL of the RNA elute was used for reverse transcription with Superscript III (Invitrogen) using the recommended reaction supplemented with 0.5 µL of RiboLock RNase Inhibitor (Thermo Scientific) and 100 pmol of NNSR-RT primer with the following protocol: 45°C 30 min; 70°C, 15 min. The cDNA was bead-purified with 1.8 volume of SPRI Beads (Beckman Coulter), eluted in 27 µL of water and subjected to RNase-H (NEB) digestion at 37°C for 30 min followed by heat inactivation. After bead purification the 20 µL cDNA elute was used for 2nd strand synthesis in a 50 µL reaction containing: 1x NEB Buffer 2, 25 nmol dNTP, 5 U of exo(-) Klenow Fragment (NEB), 200 pmol of NNSR-2 Primer for 30 min at 37°C. After bead purification

half of the DNA elute was used for a 50 μL PCR reaction containing the NEBNext High-Fidelity 2x Master Mix (NEB), 25 pmol, each, of NNSR-Illumina and NNSR-nest-ind primers with the following cycling conditions: 98°C 10 sec; 5 cycles of 98°C 10 sec, 55°C 30 sec, 72°C 30 sec; 20 cycles of 98°C 10 sec, 65°C 30 sec, 72°C 30 sec; 72°C 5 min. 15 μL of the PCR reaction was separated on a 1% agarose gel and the smear of 500-700 bp was isolated. The indexed libraries were quantified by qPCR using the NEBNext Library Quant Kit for Illumia (NEB, mixed and sequenced on a MiSeq instrument (Illumina)) with a 2x250 paired-end setup.

RNA sequencing analysis

Quality trimming and adapter removal were performed using fastp (v0.20.0 (11)). Read 1 and 2 adapter recognition sequences were provided for adapter removal (Illumina TruSeq Adapter Read 1: AGATCGGAAGAGCACACGTCTGAACTCCAGTCACNNNNNNATCTCGTATGCCGTCTTCTGCTTG, Illumina TruSeq Adapter Read 2: AGATCGGAAGAGCGTCGTGTAGGGAAAGAGTGT; NNNNNN: sample-specific index) and the leading two nucleotides were removed from each read (--trim_front1 2 --trim_front2 2). For quality trimming, bases in sliding windows with a mean quality below 30 (-5 -3 --cut_mean_quality 30) were discarded on both sides of the reads. Base correction in overlapping regions (-c) was applied. Reads with Ns and a length below-< 30 bp after trimming (-n 0 -l 30) were discarded.
Mapping was performed with BWA mem v 0.7.12-r1039 (12), using default parameters unless stated otherwise. Host-derived reads were removed by mapping quality controlled reads against the African green monkey genome (*Chlorocebus sabeus*, RefSeq assembly GCA_000409795.2), specifying the minimum seed length (-k 31). Unmapped reads were extracted using samtools v1.7 (13) and bamToFastq v2.17.0 (14), and subsequently mapped to the plasmid reference genomes of either MeV$_{vac2}$-SARS2-S(H) or MeV$_{vac2}$-SARS2-S(P), as appropriate. Host-free alignments were deduplicated using picard-tools MarkDuplicates (http://broadinstitute.github.io/picard) and left-aligned using GATK LeftAlignIndels v4.0 (15).
Sample majority consensus sequences were obtained by substituting minor frequency variants in the respective virus reference sequence for alternative variants with allele frequencies > 50%. Variant calling was performed with LoFreq v2.1.3 (16) using default parameters.

Immunoperoxidase monolayer assay (IPMA)

For immunoperoxidase monolayer assay, Vero cells cultured in flat-bottom 12-well plates were fixed overnight with methanol at −20°C two days after infection with a MOI of 0.01. The fixed cells were then washed three times with 1 mL PBS and subsequently blocked with PBS containing 2% bovine serum albumin (BSA) (Roth, Karlsruhe, Germany) for 30 min at 37°C. The cells were then probed for 1 h with a polyclonal rabbit anti-SARS-CoV-2-S protein antibody (1:2,250; ab252690; Abcam, Cambridge, UK) or a rabbit anti-MeV N protein antibody (1:1,000, ab23974, Abcam) in PBS with 2% BSA. The cells were washed 3 times with 1 mL PBS and subsequently incubated with the secondary HRP-coupled donkey anti-rabbit IgG(H+L) polyclonal antibody (1:1,000; 611-7202; Rockland, Gilbertsville, USA) for 1 h at 37°C. Then, the cells were washed 3 times, again. For detection, the cells were stained with TrueBlue peroxidase substrate solution (SeraCare, Milford, USA).

Western Blot Analysis

Cells were lysed and immunoblotted as previously described (17). Rabbit anti-SARS-S protein antibody (1:3,000; ab252690; Abcam), rabbit anti-MeV-N protein polyclonal antibody (1:5,000; ab23974; Abcam), and a mouse anti-ß-actin antibody (1:5,000; ab6276; Abcam) were used. Donkey anti-rabbit IgG-HRP (H&L) polyclonal antibody (1:10,000; 611-7202; Rockland) and goat anti-mouse IgG-HRP (1:10,000; A2554-1ML; Merck, Darmstadt, Germany) served as secondary antibodies. Peroxidase activity was visualized with an enhanced chemiluminescence detection kit (Thermo Scientific, Bremen, Germany) on ChemiDoc MP Imaging System (Biorad, Dreieich, Germany).

Animal experiments

All animal experiments were carried out in compliance with the regulations of German animal protection laws and as authorized by the RP Darmstadt in consideration of the ARRIVE guidelines. Six- to 12-week-old old, treatment-naïve IFNAR$^{-/-}$-CD46Ge mice (18) that are deficient for type I IFN receptor and transgenically express human CD46 were bred in-house under SPF conditions and regularly controlled by animal care takers and institutional veterinarians for general signs of well-being, and animal weight was additionally controlled once a week during the experiments. For the experiments, animals were randomized for age- and sex-matched groups and housed in IVC cages in groups of 3 to 5 animals with nist packs as environmental enrichment at room temperature with regular 12 h day and night intervalls. Group sizes were calculated based on statistical considerations to yield sufficient statistical power as authorized by the respective competent authority. These animals were inoculated intraperitoneally (i.p.) with 1x10^5 TCID50 of recombinant vaccine viruses in 200 μL volume, or subcutaneously (s.c.) with 10 μg recombinant SARS-CoV-2 S protein (Sino Biological Europe, Eschborn, Germany) adjuvanted with 500 μg aluminum hydroxide (Alhydrogel adjuvant 2%, vac-alu-250, InvivoGen, San Diego, CA, USA) in 100 μL volume on days 0 and 28. 200 μL blood was collected on days 0, and 28, while final serum was collected on day 49 post initial immunization (p.i.). serum samples were stored at -20°C. Mice were euthanized on day 49 p.i., and splenocytes were harvested for assessment of cellular immune responses.

For challenge experiments, 6 – 12 week old Syrian golden hamsters (Envigo RMS, Venray, Netherlands) or IFNAR$^{-/-}$-CD46Ge mice were vaccinated on days 0 and 21 as described above, but using a weight-adapted dose of 5×10^5 $TCID_{50}$ vaccine virus or 10 μg recombinant SARS-CoV-2 S protein adjuvanted with 500 μg aluminum hydroxide for the hamsters. Blood was drawn on days 0, 21 and at the day of challenge. Hamsters were challenged on day 35 applying i.n. a dose of 4×10^3 $TCID_{50}$ SARS-CoV-2 (isolate MUC-IMB1) in passage 1 in 100 μL volume. Mice were challenged on day 37 applying i.n. a dose of 1×10^5 $TCID_{50}$ SARS-CoV-2 MA (19) in passage 2 in 30 μL volume.

Total IgG and IgG1-/IgG2a quantification

MeV bulk antigens (10 μg/mL; Virion Serion, Würzburg) or recombinant SARS-CoV-2 S protein (5 μg/mL) were coated in 50 μL carbonate buffer (Na_2CO_3 30 mM; $NaHCO_3$ 70 mM; pH 9.6) per well on Nunc Maxisorp® 96 well ELISA plates (ebioscience) and incubated overnight at 4°C. The plates were washed three times with 200 μL ELISA washing buffer (PBS, 0.1% Tween 20 (w/v)) and blocked with 100 μL Blocking buffer (PBS; 5% BSA; 0.1% Tween 20) for at least 2 h at room temperature. Mouse

sera were 5-fold serially diluted in ELISA dilution buffer (PBS, 1% BSA, 0.1% Tween 20), and 50 µL/well were used for the assay. The plates were incubated at 37°C for 2 h and washed three times with ELISA washing buffer, followed by incubation with 50 µL/well of HRP-conjugated rabbit anti-mouse total IgG (1:1,000 in ELISA dilution buffer; P0260, Dako Agilent, Santa Clara, CA, USA), goat-anti-mouse IgG1 (1:8,000 in ELISA dilution buffer; ab97240, Abcam, Cambridge, UK), or goat-anti-mouse IgG2a (1:8,000 in ELISA dilution buffer; ab97245, Abcam) at room temperature for 1 h. Subsequently, the plates were washed four times and 100 µL TMB substrate (ebioscience) was added per well. The reaction was stopped by addition of 50 µL/well H_2SO_4 (1 N) and the absorbance at 450 nm (specific signal) and 630 nm (reference wavelength) was measured.

Th1/Th2 cytokine multiplex assay

Quantification of Th1/Th2 cytokines in supernatant of splenocytes was performed using mouse high sensitivity T cell magnetic bead panel assay (MHSTCMAG-70K, Merck, Darmstadt, Germany). 5×10^5 isolated splenocytes were co-cultured with different stimuli in 200 µL RPMI + 10% FBS, 2 mM L-Gln, and 1% penicillin-streptomycin for 36 h. For re-stimulation of SARS-CoV-2 S protein-specific T cells, splenocytes were co-cultivated with 5×10^4 DC2.4 dendritic cells, the corresponding cell line transgenically expressing SARS-CoV-2 S protein or medium alone. After 36 h, cells were spun down and supernatants were collected and stored at -20°C until assayed. For multiplex assay, cytokines were coupled over night to magnetic beads coated with capture antibodies, labeled with biotinylated detection antibody and incubated with Streptavidin-PE conjugate. Fluorescence was measured using MAGPIX with xPONENT software (Luminex Instruments, Thermo Scientific, Bremen, Germany).

Virus neutralization test (VNT)

Virus neutralizing titers (VNT) were quantified as described previously (3). Towards this, sera were serially diluted in 2-fold dilution steps in DMEM in duplicates. A total of 50 PFU of MV_{vac2}-GFP(P) or 100 $TCID_{50}$ of SARS-CoV-2 (isolate MUC-IMB1) were mixed with diluted sera and incubated at 37°C for 1 h. MeV or SARS-CoV-2 virus-serum suspensions were added to 1×10^4 Vero or Vero E6 cells, respectively, seeded 4 h prior to the assay in 96-well plates and incubated for 4 days at 37°C. VNTs were calculated as the reciprocal of the highest mean dilution that abolished infection.

Plaque reduction neutralization test ($PRNT_{50}$)

Plaque reduction neutralizing titers ($PRNT_{50}$) were determined as follows: Triplicates of sera were 2-fold serially diluted in DMEM, and 50 µL DMEM containing 100 $TCID_{50}$ SARS-CoV-2 was added per well and incubated at 37°C for 1 h. Then, the virus-serum mixture was added to 8×10^5 Vero cells seeded in a 6-well plates 24 h before and incubated for 1 h at 37°C while rocking plates every 15 min. After removal of the inoculum, cells were overlaid with 1.5% Avicel RC-591NF (FMC BioPolymer, Co. Cork, Ireland) in 2 ml complete DMEM. For assay readout 3 days after infection, cells were fixed with 4% formalin in PBS and stained with 0.1% crystal violet. $PRNT_{50}$ was determined as the reciprocal dilution leading to at least 50% reduction in plaque numbers relative to the mean of serum dilutions of mock mice.

IFN-γ ELISpot Analysis

Murine interferon gamma (IFN-γ) enzyme-linked immunosorbent spot (ELISpot) assays were performed using the Mouse IFN-γ ELISPOT Pair kit including capture and detection antibody (BD Bioscience,

Franklin Lakes, NJ, USA) and HRP Streptavidin (BD Bioscience) for ELISpot detection in combination with multiscreen immunoprecipitation (IP) ELISpot polyvinylidene difluoride (PVDF) 96-well plates (Merck Millipore, Darmstadt, Germany) according to the manufacturer's instructions. 5×10^5 isolated splenocytes were co-cultured with different stimuli in 200 μL RPMI + 10% FBS, 2 mM L-Gln, and 1% penicillin-streptomycin for 36 h. For re-stimulation of SARS-CoV-2 S protein-specific T cells, splenocytes were co-cultivated with 5×10^4 JAWSII, DC2.4 dendritic cells, or the corresponding cell lines transgenically expressing SARS-CoV-2 S protein. In parallel, splenocytes were stimulated with 10 μg/mL MeV bulk antigen (Virion Serion). For general T cell stimulation, 10 μg/mL concanavalin A (ConA, Sigma-Aldrich) was used, and as negative control, splenocytes were left untreated. After 36 h, cells were spun down, supernatants were removed, and cells were lysed in the wells by hypotonic shock. Plates were incubated with biotin-conjugated anti-IFN-γ detection antibodies and streptavidin-HRP according to the manufacturer's instructions. 3-Amino-9-ethyl-carbazole (AEC; Sigma-Aldrich) was dissolved in N,N-dimethylformamide (Merck Millipore) and used for peroxidase-dependent staining. Spots were counted using an Eli.Scan ELISpot scanner (AE.L.VIS, Hamburg, Germany) and ELISpot analysis software Eli.Analyse V5.0 (AE.L.VIS).

Intracellular cytokine staining

For flow cytometry-based analysis of cytokine expression by intracellular cytokine staining (ICS), splenocytes of vaccinated mice were isolated, and 2×10^6 splenocytes per mouse were cultivated in 200 μL RPMI1640 + 10% FBS, 2 mM L-Gln, 1× non-essential amino acids (Biochrom), 10 mM HEPES, 1% penicillin-streptomycin, 50 μM β-mercaptoethanol, 10 μg/mL brefeldin A (Sigma-Aldrich) with DC2.4-SARS2-S cells as used for ELISpot analysis. For general T cell stimulation, 0.25 μg/mL tetradecanoylphorbol acetate (TPA, Sigma Aldrich) and 0.5 μg/mL ionomycin (Iono, Sigma-Aldrich) were used as positive control, and medium alone served as negative control. Splenocytes were stimulated for 5 h at 37°C. Subsequently, cells were stained with fixable viability dye eFluor450 (eBioscience), α-CD4-PE (1:2,000; Cat.-No. 553049 BD, Franklin Lakes, NJ, USA), α-CD8-FITC (1:500; Cat.-No. 553031, BD), and α-CD3-PerCPCy5.5 (1:500; Cat.-No. 550763, BD). Subsequent to permeabilization with Fixation/Permeabilization Solution (BD) and Perm/Wash Buffer (BD), cells were stained with α-IFN-γ-APC (1:500; Cat.-No. 554413, BD), α-IL-2-AlexaFluor700 (1:200; Cat.-No. 503818, Biolegend, San Diego, USA) and α-TNF-α-Pe-Cy7 (1:500; Cat.-No. 557644, BD). Cells were fixed with ice-cold 1% paraformaldehyde (PFA) in PBS and analyzed via flow cytometry using an LSRII SORP flow cytometer (BD) and DIVA software (BD).

T cell proliferation assay

Splenocytes isolated three weeks after the second immunization were labeled with 0.5 μM carboxyfluorescein-succinimidyl-ester (CFSE) (ebioscience, Life Technologies, Carlsbad, CA, USA) as previously described (20). In brief, 5×10^5 labelled cells were seeded in RPMI 1640 supplemented with 10% mouse serum, 2 mM L-Glutamine, 10 mM HEPES, 1% penicillin/streptomycin, and 100 μM 2-mercaptoethanol in 96-wells. 200 μL Medium containing 10 μg/ml Concanavalin A (Con A, Sigma-Aldrich), 10 μg/mL MeV bulk antigen (Virion Serion), or 5×10^3 DC2.4-SARS2-S cells were added to each well, and cultured for 6 d. Medium and wild type DC2.4 and JAWSII cells served as controls. Stimulated

cells were subsequently stained with α-CD3-PacBlue (1:50; clone 500A2; Invitrogen Life Technologies), α-CD8-APC (1:100; clone 53-6.7; ebioscience) and α-CD4-PE (1:2000; Cat. 553049; BD) antibodies and fixed with 1% PFA in PBS. Finally, the stained cells were analyzed by flow cytometry using an LSR II flow cytometer (BD) and FCS Express software (De Novo Software).

CTL killing assay

For re-stimulation of T cells isolated 3 weeks after the second immunization, $5×10^6$ splenocytes were co-cultured with $5×10^4$ DC2.4-SARS2-S cells for 6 days in 12-wells in RPMI 1640 supplemented with 10% FBS, 2 nM L-Glutamin, 1 mM HEPES, 1% penicillin/streptomycin, 100 μM 2-mercaptoethanol, and 100 U/ml murine rIL-2 (Peprotech, Hamburg, Germany). $5×10^3$ EL-4_{red} cells were labeled with 0.5 μM CFSE and mixed with $5×10^3$ EL-4_{green}-SARS2-S cells per well. Splenocytes were counted and co-cultured with EL-4 target cells at the indicated ratios for 4 h at 37°C. Afterwards, EL-4 cells were labeled with Fixable Viability Dye eFluor® 780 (ebioscience), fixed with 1% paraformaldehyde (PFA), and analyzed by flow cytometry using an LSR II flow cytometer (BD) and FCS Express. For indication of Antigen:NC EL-4 ratio the cell count of viable SARS-CoV-2 S-expressing cells was divided by the population of viable negative controls.

Determination of virus titers in organs of infected animals

Organs were snap-frozen in liquid nitrogen and were subsequently homogenized in 1 ml cold Titration Medium (Hanks' Balanced Salt solution containing 10% glycerol, 5 mg/mL lactalbumin, 304 μg/mL Penicilin, 500 μg/mL Streptomycin, 250 μg/mL Gentamicin, 50 U/mL Nystatin) using Lysing Matrix M tubes (MP Bio) and the TissueLyser LT (QIAGEN) for 2x 1 min at 50 Hz and were kept on ice before, between and after homogenization. Vero E6 cells were inoculated with 10-fold serially diluted organ homogenates and cultivated for 4 d at 37°C. The SARS-CoV-2 organ titer was calculated by the $TCID_{50}$ method of Kaerber and Spearman according to the virus-induced CPE and normalized to 1 g of homogenized tissue.

RNA preparation For purification of viral RNA from hamster or mouse tissue, 25 mg frozen nasal turbinates or 90 mg of lung tissue were homogenized in 1 mL TRIzol Reagent (Ambion, Thermo Fisher Scientific) in Lysing Matrix M tubes (MP Bioscience, Hilton, UK) using the TissueLyser LT (QIAGEN) for 1x 1 min and 1x 2 min at 50 Hz while keeping samples on ice before, between and after homogenization. To remove organ debris, homogenates were centrifuged (10 min, 6,800 rpm, 4°C) and the supernatant was subsequently used for RNA purification with the Direct-zol RNA MiniPrep kit (Zymo research, Freiburg (Breisgau), Germany) according to manufacturers instructions and RNA was eluted in 50 μL RNAse-free water.

Determination of virus genome copy numbers by qRT-PCR

SARS-CoV-2 or SARS-CoV-2 MA RNA, was quantified via multiplex quantitative reverse transcription-PCR (qRT-PCR) using Superscript III one step RT-PCR system with Platinum Tag Polymerase (Invitrogen, Darmstadt, Germany). Primer and probe sequences for the E gene were used as described (21). Reactions were performed in 96-wells with 5 μL of RNA in a total volume of 25 μL run in triplicates on a CFX 96 qPCR cycler (Bio-Rad Laboratories, Hercules, CA) . For analysis of samples, the NIBSC

reference standard (NIBSC 19/304) of SARS-2-CoV RNA was spiked with hamster or mouse RNA and purified as described. For mouse samples, this standard was used directly (linear range, 2.5×10^5 to 2.5×10^2 copies). For hamster samples, this standard was used for validation of an internal hamster reference used for quantification in subsequent runs (linear range, 4.5×10^6 to 4.5×10^2 copies). The cycling conditions were as follows: reverse transcription for 600 sec at 55°C, followed by denaturation for 180 sec at 94°C, and 45 cycles of 15 sec at 94°C and 30 sec at 58°C. Quantified sample copy numbers were normalized to mg of tissue used for preparation of 5 μL RNA.

Statistical analyses

To compare the means of different groups in growth kinetics, a non-parametric One-way ANOVA was performed. For ICS analysis, the non-parametric two-tailed Mann-Whitney test was used to compare cytokines levels between DC2.4 and DC2.4-SARS2-S- restimulated splenoycytes within the MeV_{vac2}-SARS2-S(H) vaccine group. Note, that these exploratory analyses have been done without correction for multiple testing. For proliferation assay the mean differences were calculated and analyzed using one-tailed Mann-Whitney t-test. To all three groups in CTL killing assays a linear curve was fitted for antigen vs. logarithmised effector-target ratio E:T. The p values testing for differences in slopes were calculated and populations of SARS2-S(H) compared with control ATU vaccinated cells. The p values were not adjusted for multiplicity due to the explorative character of the study. For VNT, fusion activity, organ titer, and copy number statistical analysis, one-way ANOVA was performed in combination with Tukey's Multi comparison test to compare all pair means. For comparison of mouse copy number data between two groups, unpaired two-tailed t-test was applied. For multiplex statistical analysis, two-way ANOVA analysis was applied with paired Tukey's Multi comparison test as post hoc test. For statistical analysis of grouped ELISpot data, two-way ANOVA analysis was applied with paired Tukey's Multi comparison test.

Supplementary Figures

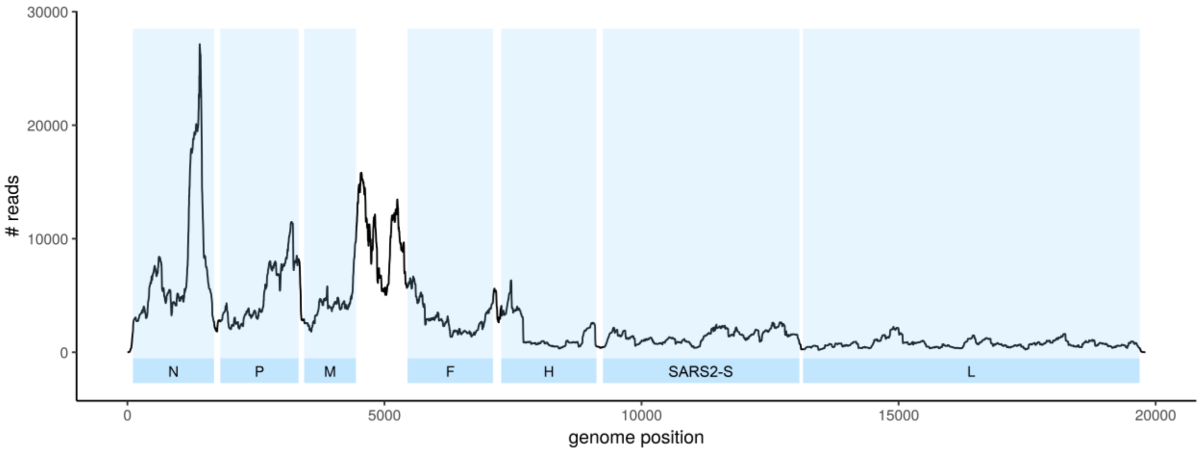

Fig. S1: Coverage of vaccine candidate MeV$_{vac2}$-SARS2-S(H) genome during next generation sequencing. Schematic depiction of read frequency at each position of the vaccine virus genome. Blue areas indicate respective viral coding sequences, white areas indicate intergenic regions and untranscribed terminal regions (UTRs) of the genome. Coverage across the genome was sufficient for variant detection and reflects the transcription gradient typically observed in measles virus total RNAseq data. Since the majority of reads are mRNA-derived, low read numbers decrease strongly between the coding regions and continually towards the 5' end.

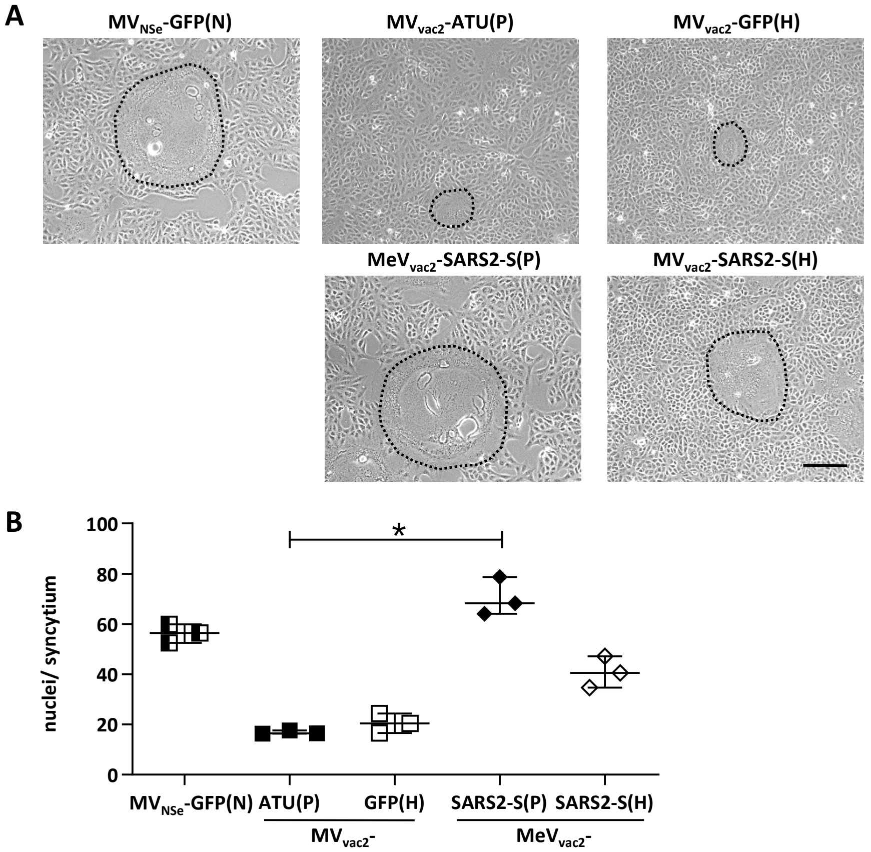

Fig. S2: Characterization of fusogenic phenotype of MeV_{vac2}-SARS2-S(P) and MeV_{vac2}-SARS2-S(H). (A) Photographs of fusion activity of Vero cells infected at an MOI of 0.01 with MeV_{vac2}-SARS2-S(P) or MeV_{vac2}-SARS2-S(H) encoding SARS-CoV-2 S in additional transcription units post-P or post-H, respectively, in direct comparison to MV_{vac2}-ATU(P) or MV_{vac2}-GFP(H) control vaccine viruses or MV_{NSe}-GFP(N) hyperfusogenic oncolytic MeV. Representative picture of one out of three independent experiments. Scale bar represents 200 µm. **(B)** Cell fusion was quantified 30 h after infection. For statistical analysis, one-way ANOVA was performed in combination with Tukey's Multi comparison test to compare all pair means. *, $p<0.05$.

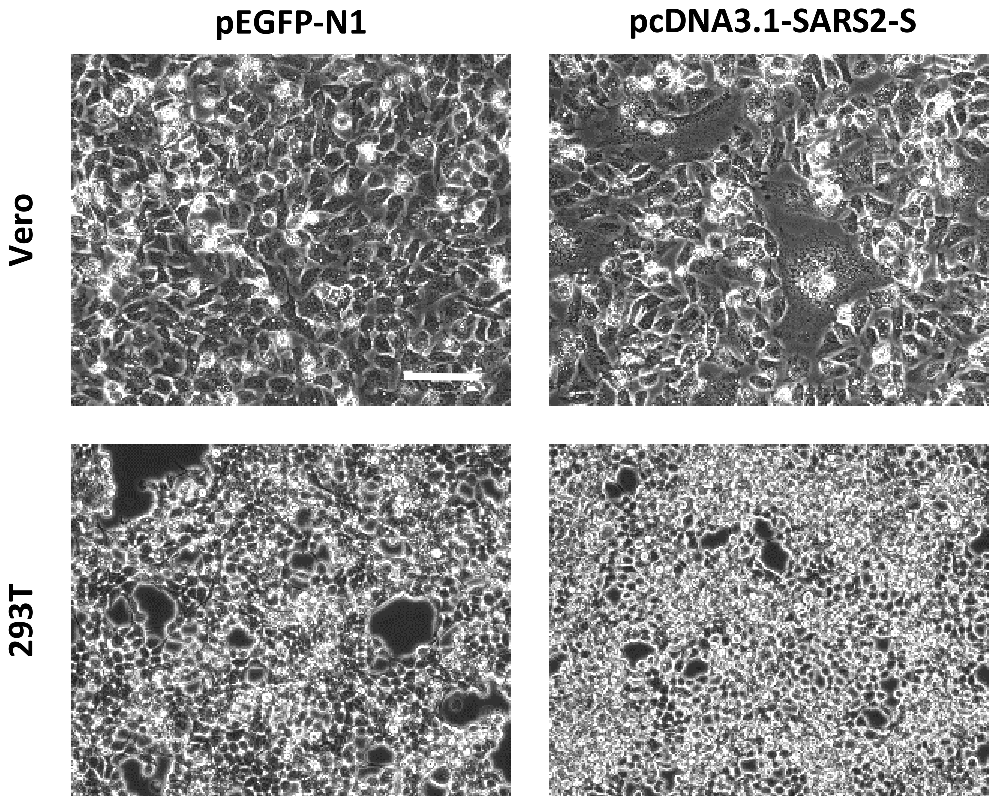

Fig. S3: Expression of SARS-CoV-2 S protein in Vero and 293T cells. Photographic depiction of fusion activity in Vero or 293T cells 48 h after transfection with 1 µg of SARS-CoV-2 S expression plasmid or control DNA. One representative out of three independent experiments is shown. Scale bar represents 100 µm.

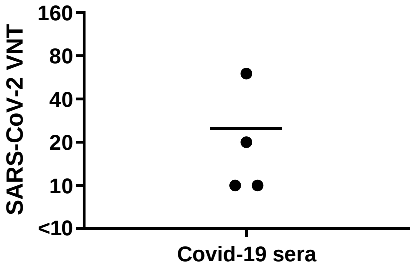

Fig. S4: SARS-CoV-2 VNT in four human COVID-19 reconvalescent patient sera. Virus neutralizing titers (VNT) were calculated as reciprocal of the highest dilution abolishing infectivity. Selected human patient sera were tested with the same assay conditions as mouse or hamster sera. Dots represent single individuals; horizontal line represents mean.

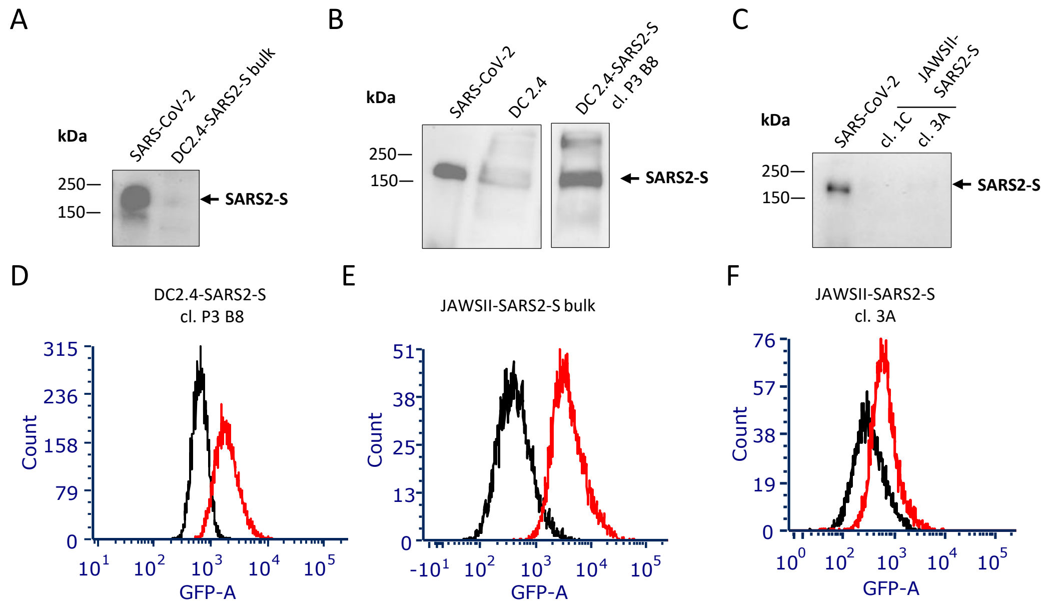

Fig. S5: Antigen expression in transduced DCs. **(A-C)** Immunoblot analysis of SARS-CoV-2 Spike (S) antigen expression in **(A)** DC2.4-SARS2-S bulk **(B)** the single cell clone P3 B8 of DC2.4-SARS2-S used for stimulation experiments and **(C)** two selected single cell clones of JAWSII-SARS2-S dendritic cell cultures stably transduced with lentiviral expression vectors encoding SARS-CoV-2 Spike glycoprotein as indicated. SARS-CoV-2, lysate of Vero cells infected by SARS-CoV-2. **(D-E)** flow cytometric analysis of GFP expression coupled via an IRES element on the bicistronic mRNA to the SARS-CoV-2 Spike GP ORF of **(D)** single cell clone P3 B8 of DC2.4-SARS2-S, **(E)** sorted JAWSII-SARS2-S bulk culture, and **(F)** single cell clone 3A of JAWSII-SARS2-S also depicted in (C). Black, parental cell line; red, transduced population.

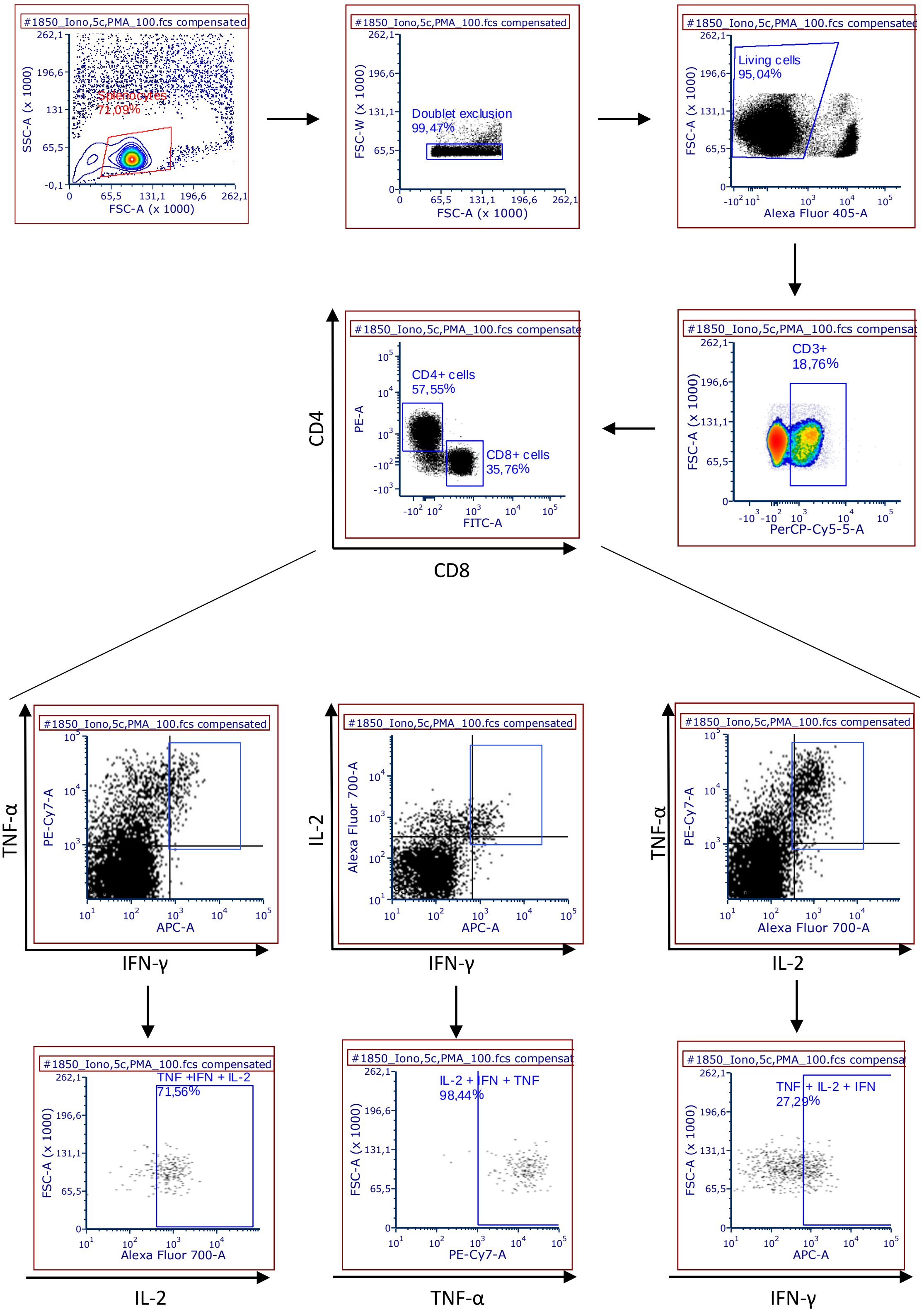

Fig. S6: Gating strategy for intracellular cytokine staining. Exemplary depiction of the gating strategy to analyze T cells after re-stimulation and staining for cytokine induction. The gating strategy includes cell doublet exclusion, selection for living cells and separation of $CD8^+$ and $CD4^+$ T cells within $CD3^+$ splenocyte populations. Respectively gated T cell populations were then analyzed for expression of IFN-γ, TNF-α, or IL-2. Multi-colour flow cytometry allows assessment of double- or triple-positive cells, exemplarily shown for $CD4^+$ T cells after stimulation with ionomycin and PMA.

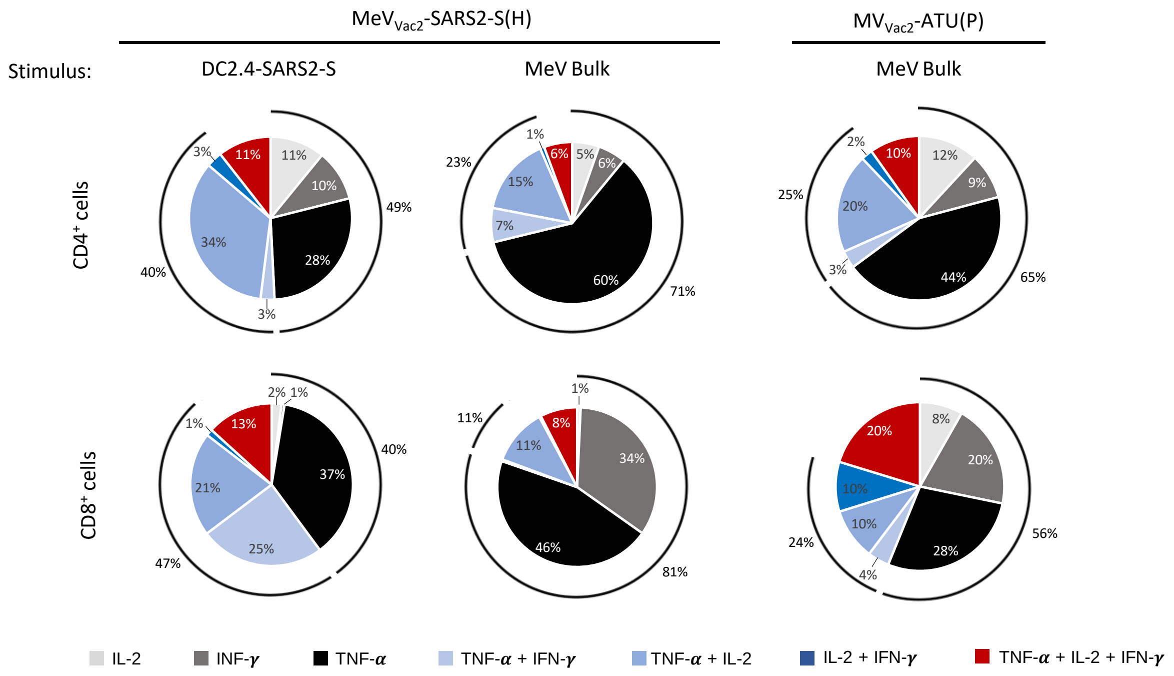

Fig. S7: Multi-functionality of induced T cell responses. Depicted are pie-charts of T-cell responses induced by vaccination with MeV$_{vac2}$-SARS2-S(H) or MV$_{vac2}$-ATU(P) upon antigen-specific re-stimulation. Data as depicted in Fig. 4 were analyzed for co-expression of the different cytokines. Poly-functional T cells revealed as fractions of cell populations expressing one, two, or all three of the tested cytokines and indicating the size of each fraction among all responsive T cells.

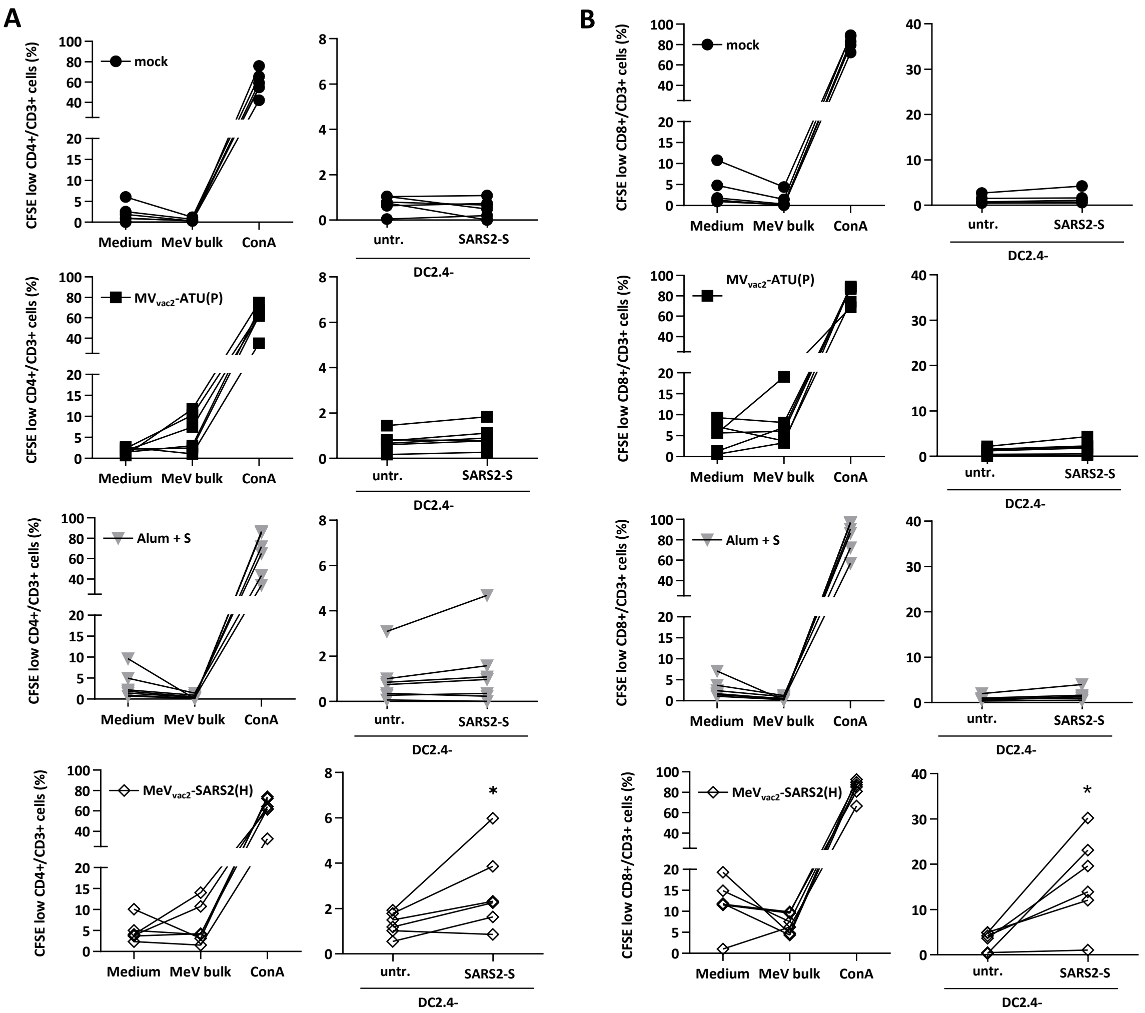

Fig. S8: Ag-specific proliferation of SARS-CoV-2 S-specific T cells. Proliferation assay using splenocytes of mice vaccinated on days 0 and 28 with indicated viruses, isolated 21 days after boost immunization, after co-culture with DC2.4 dendritic cell line transgenic for SARS-CoV-2 S (SARS2-S) or untransduced controls (untr.). Depicted are the percentages of **(A)** $CD4^+$ or **(B)** $CD8^+$ T cells with low CFSE staining, indicating proliferation in the samples. To analyze cellular α-MeV responses, splenocytes were stimulated with 10 μg/mL MeV bulk antigens or were left unstimulated (medium). The reactivity of splenocytes was confirmed by Concanavalin A (ConA) treatment (10 μg/mL). Results for splenocytes of vaccinated mice are displayed individually and the trend between paired unstimulated and re-stimulated samples is outlined (n = 6-7). One-tailed Mann-Whitney t-test. *, $p<0.05$.

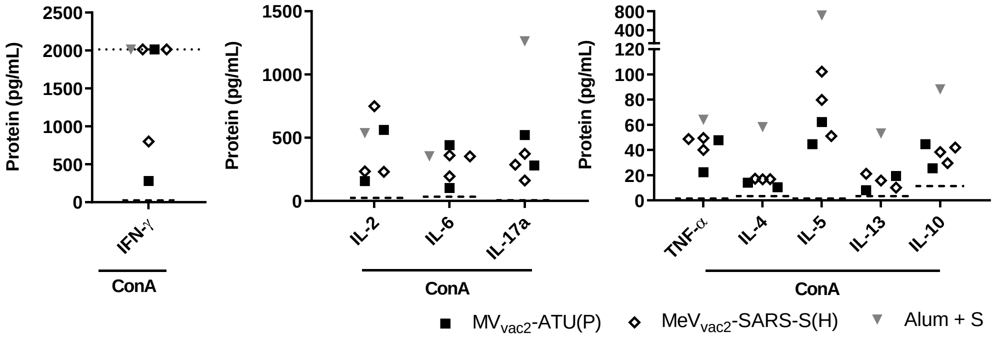

Fig. S9: Cytokine-expression by ConA-stimulated splenocytes. General reactivity of splenocytes used in multiplex cytokine assay (s. Fig. 6B) were demonstrated by Concanavalin A (ConA) treatment (10 µg/mL). Splenocytes of representative mice vaccinated with MV_{vac2}-ATU(P), MeV_{vac2}-SARS2-S(H) or Alum-adjuvanted S protein as indicated were analyzed by multiplex cytokine analysis for secretion of typical marker cytokines in the supernatant after re-stimulation. Dots represent individual animals. Dashed line: Median cytokine secretion of unstimulated splenocytes (medium, s. Fig. 6B) of all mice. Upper limit of detection: IFN-g: 2015.2 pg/mL (dotted line), IL-2: 4250.9 pg/mL; IL-6: 3992.4 pg/mL; IL-17a: 2108.5 pg/mL; TNF-a: 1408.1 pg/mL; IL-4: 408.4 pg/mL; IL-5: 4051.4 pg/mL; IL-13: 14196 pg/mL; IL-10: 5480.1 pg/mL.

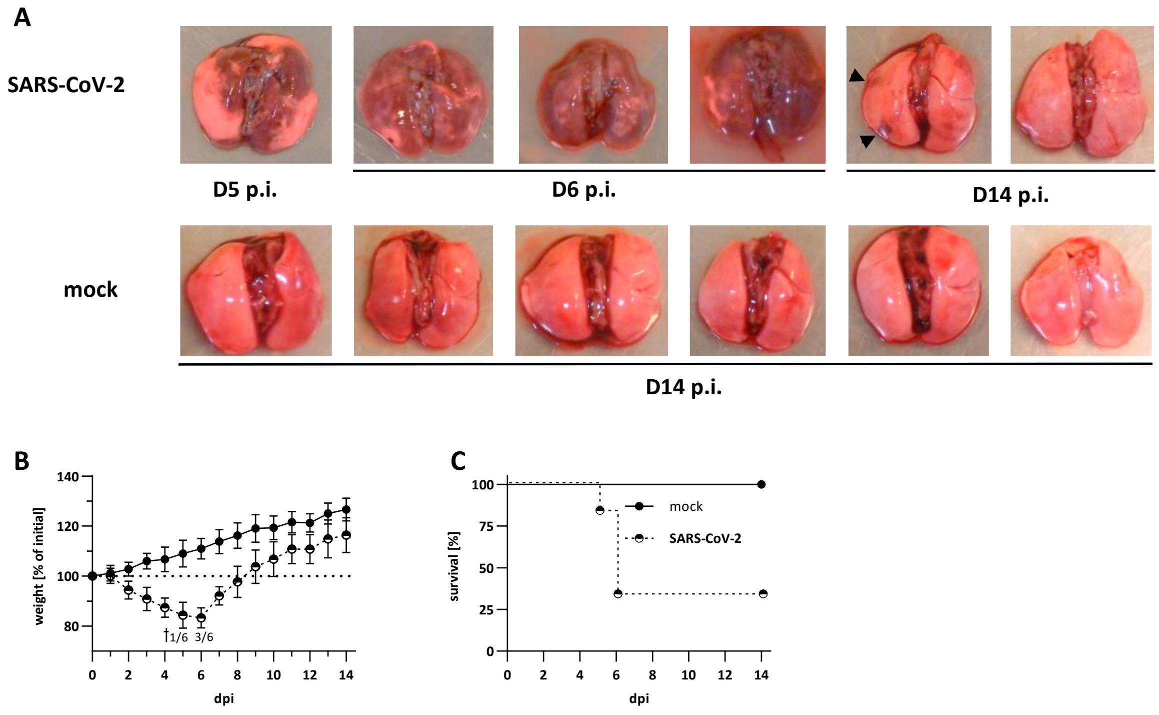

Fig. S10: SARS-CoV-2 hamster challenge model. **(A)** Macroscopic pictures of Syrian hamster lungs after SARS-CoV-2 or mock infection. Pictures were taken at the day of necropsy (20% weight loss) or at the end of the experiment 14 dpi, as indicated. Arrows point at macroscopic lesions still visible in one lung 14 dpi. **(B)** Body weight changes of SARS-CoV-2- and mock-infected Syrian hamsters (n = 6). **(C)** Kaplan-Meier plot of hamster survival following SARS-CoV-2 or mock infection.

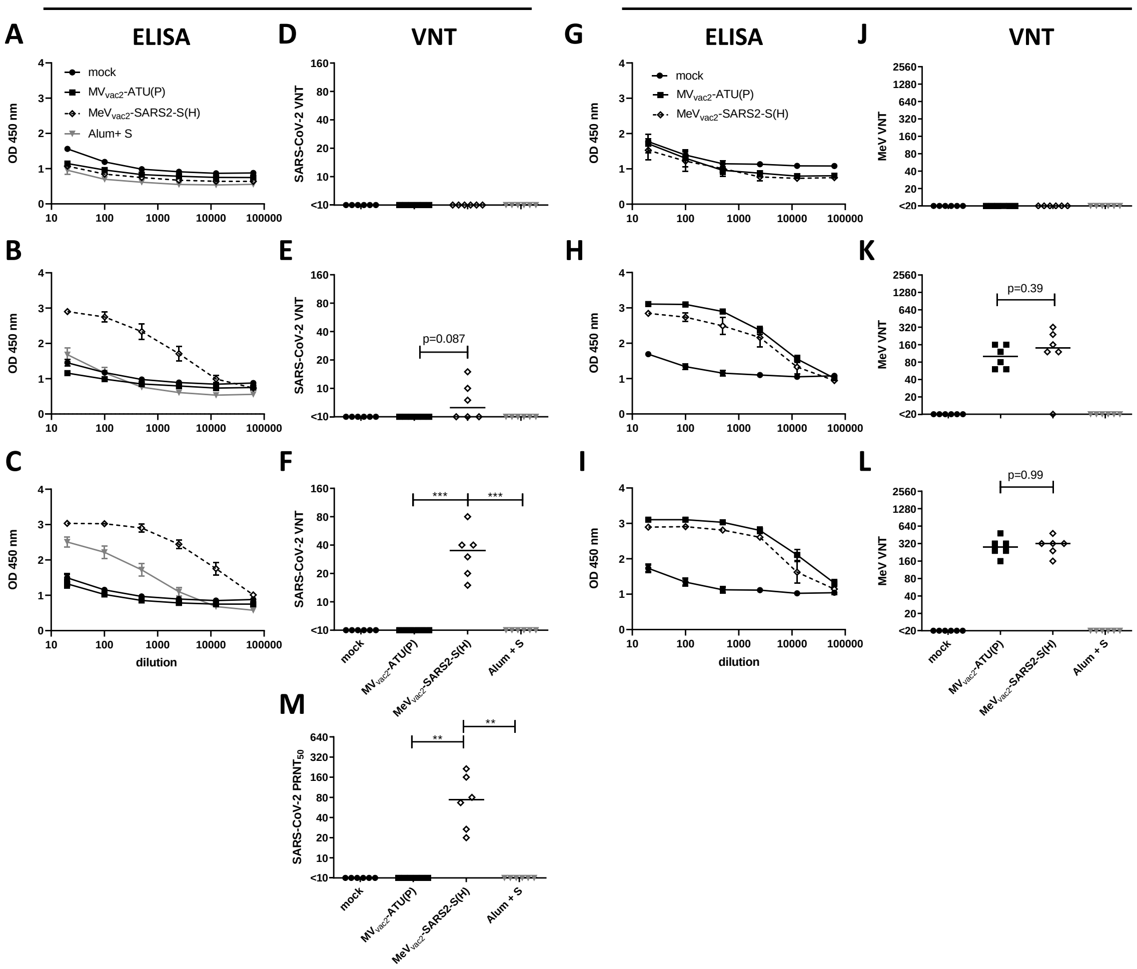

Fig. S11: Humoral immunity in vaccinated hamsters challenged with SARS-CoV-2. Sera of Syrian hamsters vaccinated on days 0 and 21 with indicated viruses or Alum-adjuvanted S protein were sampled on day 0 **(A, D, G, J)**, day 21 after prime- **(B, E, H, K)** and day 35 after boost-immunization **(C, F, I, L)** and analyzed for antibodies specific for SARS-CoV-2 S or MeV. Medium-inoculated hamsters served as mock control. Pan-IgG binding to recombinant SARS-CoV S **(A – C)** or MeV bulk antigens (**G – I**) were determined by ELISA via the specific OD 450 nm value. Depicted are means and respective standard deviation of the mean (SEM) of each group (n = 6). Virus neutralizing titers (VNT) in vaccinated hamsters for SARS-CoV-2 **(D – F)** or MeV **(J – L)** were calculated as reciprocal of the highest dilution abolishing infectivity. Dots represent single individuals; horizontal line represents median per group. All hamsters below detection limit had no detectable VNT. **(M)** Functional SARS-CoV-2 neutralizing antibodies (nAb) were determined in the post boost sera via plaque reduction neutralization assay of SARS-CoV-2 ($PRNT_{50}$) on Vero cells. For statistical analysis of VNT and $PRNT_{50}$ data, one-way ANOVA was performed in combination with Tukey's Multi comparison test to compare all pair medians.

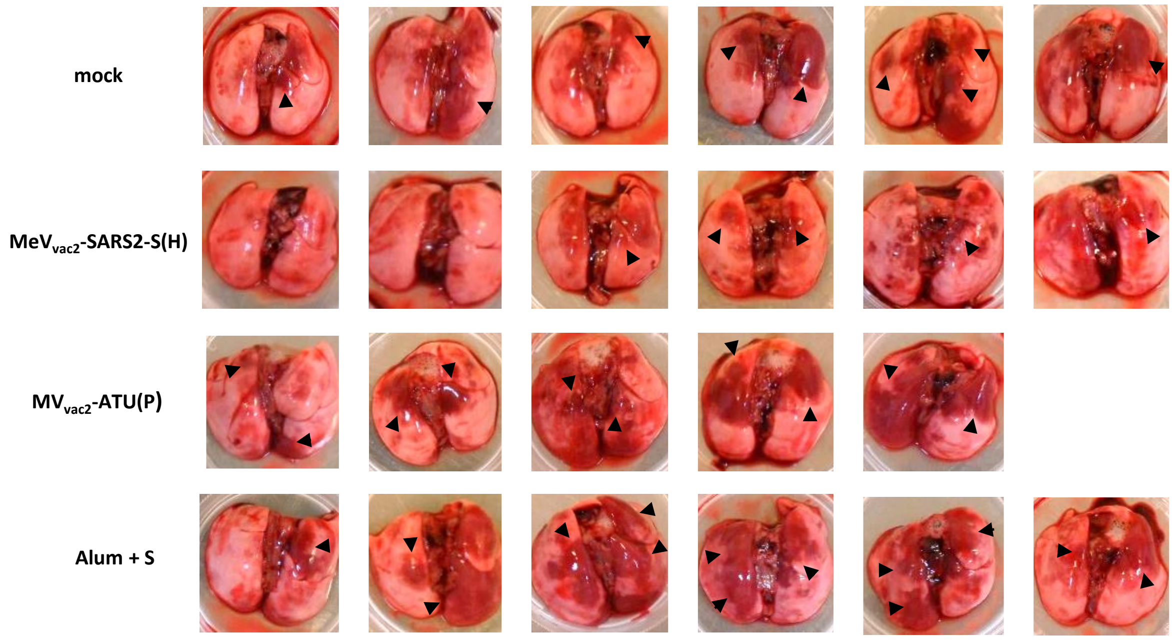

Fig. S12: Gross pathology in lungs of Syrian golden hamsters challenged with SARS-CoV-2. Hamsters were vaccinated on days 0 and 21 with indicated viruses or Alum-adjuvanted S protein and challenged at day 35 with SARS-CoV-2. Pictures were taken at necropsy on day 4 pi (n = 5 - 6). Arrow heads point at macroscopic lesions in infected lungs.

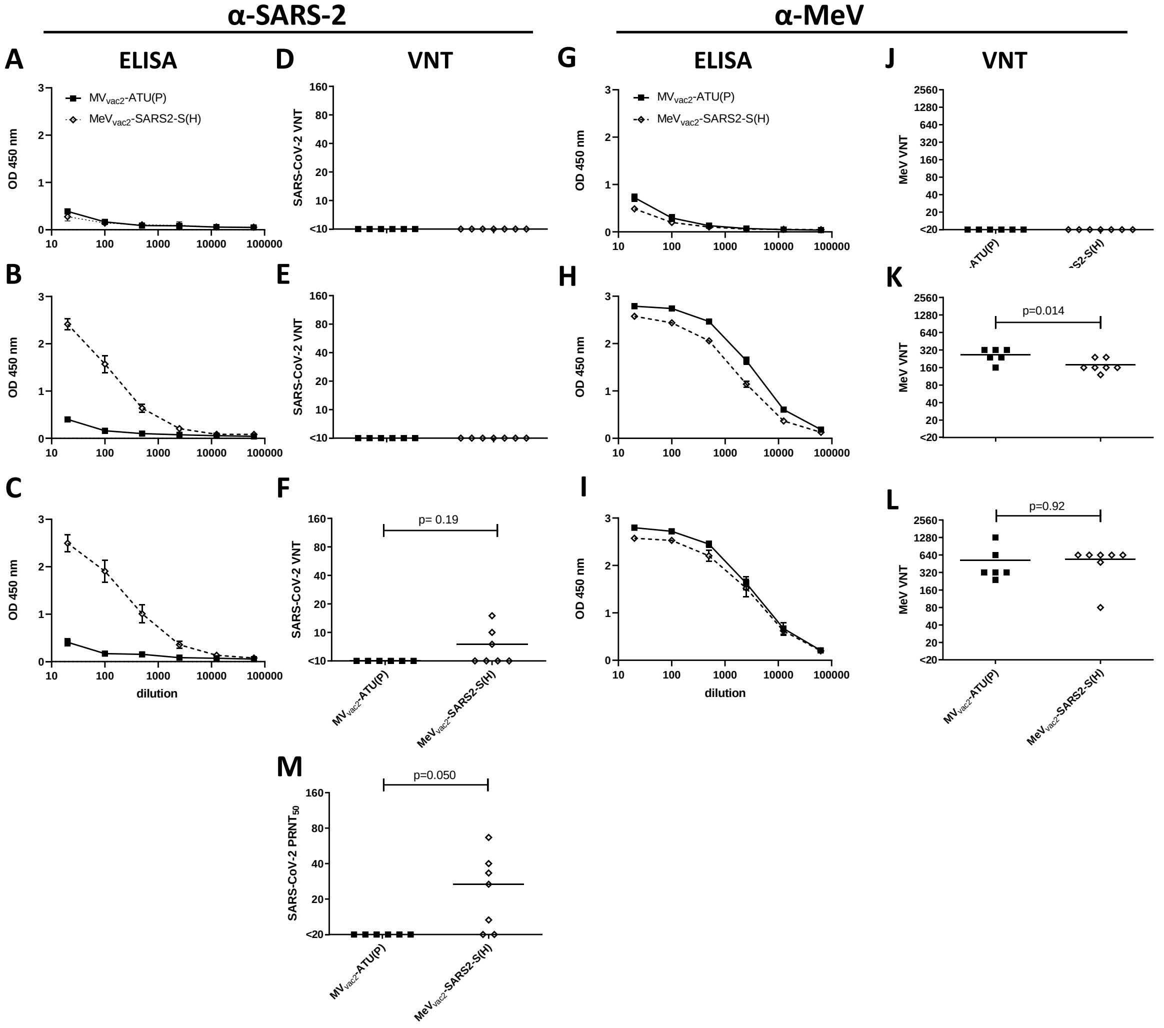

Fig. S13: Humoral immunity in mice challenged with SARS-CoV-2 MA. Sera of mice vaccinated on days 0 and 21 with indicated viruses or Alum-adjuvanted S protein were sampled on day 0 **(A, D, G, J)**, day 21 after prime- **(B, E, H, K)** and day 35 after boost-immunization **(C, F, I, L)** and analyzed for antibodies specific for SARS-CoV-2 S or MeV. Medium-inoculated mice served as mock. Pan-IgG binding to recombinant SARS-CoV-2 S **(A – C)** or MeV bulk antigens **(G - I)** were determined by ELISA via the specific OD 450 nm value. Depicted are means and respective standard deviation of the mean (SEM) of each group (n = 6 - 7). Virus neutralizing titers (VNT) in vaccinated mice for SARS-CoV-2 **(D - F)** or MeV **(J – L)** were calculated as reciprocal of the highest dilution abolishing infectivity. Dots represent single individuals; horizontal line represents mean per group. All mice depicted on the detection limit had no detectable VNT. **(M)** Functional SARS-CoV-2 neutralizing antibodies (nAb) were determined in the post boost sera via plaque reduction neutralization assay of SARS-CoV-2 ($PRNT_{50}$) on Vero cells. For statistical analysis of VNT and $PRNT_{50}$, one-way ANOVA was performed in combination with Tukey’s Multi comparison test to compare all pair medians.

SI References

1. Z. Shen, G. Reznikoff, G. Dranoff, K. L. Rock, Cloned dendritic cells can present exogenous antigens on both MHC class I and class II molecules. *Journal of immunology (Baltimore, Md. : 1950)* **158**, 2723–2730 (1997).
2. K. Friedrich, J. R. Hanauer, S. Prüfer, R. C. Münch, I. Völker, C. Filippis, C. Jost, K.-M. Hanschmann, R. Cattaneo, K.-W. Peng, A. Plückthun, C. J. Buchholz, K. Cichutek, M. D. Mühlebach, DARPin-targeting of measles virus: unique bispecificity, effective oncolysis, and enhanced safety. *Molecular therapy : the journal of the American Society of Gene Therapy* **21**, 849–859 (2013).
3. A. H. Malczyk, A. Kupke, S. Prüfer, V. A. Scheuplein, S. Hutzler, D. Kreuz, T. Beissert, S. Bauer, S. Hubich-Rau, C. Tondera, H. S. Eldin, J. Schmidt, J. Vergara-Alert, Y. Süzer, J. Seifried, K.-M. Hanschmann, U. Kalinke, S. Herold, U. Sahin, K. Cichutek, Z. Waibler, M. Eickmann, S. Becker, M. D. Mühlebach, A Highly Immunogenic and Protective Middle East Respiratory Syndrome Coronavirus Vaccine Based on a Recombinant Measles Virus Vaccine Platform. *Journal of virology* **89**, 11654–11667 (2015).
4. J. W. Hewett, B. Tannous, B. P. Niland, F. C. Nery, J. Zeng, Y. Li, X. O. Breakefield, Mutant torsinA interferes with protein processing through the secretory pathway in DYT1 dystonia cells. *Proceedings of the National Academy of Sciences of the United States of America* **104**, 7271–7276 (2007).
5. R. Zufferey, D. Nagy, R. J. Mandel, L. Naldini, D. Trono, Multiply attenuated lentiviral vector achieves efficient gene delivery in vivo (1997).
6. R. C. Münch, M. D. Mühlebach, T. Schaser, S. Kneissl, C. Jost, A. Plückthun, K. Cichutek, C. J. Buchholz, DARPins: an efficient targeting domain for lentiviral vectors. *Molecular therapy : the journal of the American Society of Gene Therapy* **19**, 686–693 (2011).
7. A. Martin, P. Staeheli, U. Schneider, RNA polymerase II-controlled expression of antigenomic RNA enhances the rescue efficacies of two different members of the Mononegavirales independently of the site of viral genome replication. *Journal of virology* **80**, 5708–5715 (2006).
8. G. Kaerber, Beitrag zur kollektiven Behandlung pharmakologischer Reihenversuche (1931).
9. J. R. del Valle, P. Devaux, G. Hodge, N. J. Wegner, M. B. McChesney, R. Cattaneo, A vectored measles virus induces hepatitis B surface antigen antibodies while protecting macaques against measles virus challenge. *Journal of virology* **81**, 10597–10605 (2007).
10. M. M. Böhmer, U. Buchholz, V. M. Corman, M. Hoch, K. Katz, D. V. Marosevic, S. Böhm, T. Woudenberg, N. Ackermann, R. Konrad, U. Eberle, B. Treis, A. Dangel, K. Bengs, V. Fingerle, A. Berger, S. Hörmansdorfer, S. Ippisch, B. Wicklein, A. Grahl, K. Pörtner, N. Muller, N. Zeitlmann, T. S. Boender, W. Cai, A. Reich, M. an der Heiden, U. Rexroth, O. Hamouda, J. Schneider, T. Veith, B. Mühlemann, R. Wölfel, M. Antwerpen, M. Walter, U. Protzer, B. Liebl, W. Haas, A. Sing, C. Drosten, A. Zapf, Investigation of a COVID-19 outbreak in Germany resulting from a single travel-associated primary case: a case series. *The Lancet Infectious Diseases* **20**, 920–928 (2020).
11. S. Chen, Y. Zhou, Y. Chen, J. Gu, fastp: an ultra-fast all-in-one FASTQ preprocessor. *Bioinformatics (Oxford, England)* **34**, i884-i890 (2018).

12. H. Li, R. Durbin, Fast and accurate short read alignment with Burrows-Wheeler transform. *Bioinformatics (Oxford, England)* **25**, 1754–1760 (2009).

13. H. Li, B. Handsaker, A. Wysoker, T. Fennell, J. Ruan, N. Homer, G. Marth, G. Abecasis, R. Durbin, The Sequence Alignment/Map format and SAMtools. *Bioinformatics (Oxford, England)* **25**, 2078–2079 (2009).

14. A. R. Quinlan, I. M. Hall, BEDTools: a flexible suite of utilities for comparing genomic features. *Bioinformatics (Oxford, England)* **26**, 841–842 (2010).

15. A. McKenna, M. Hanna, E. Banks, A. Sivachenko, K. Cibulskis, A. Kernytsky, K. Garimella, D. Altshuler, S. Gabriel, M. Daly, M. A. DePristo, The Genome Analysis Toolkit: a MapReduce framework for analyzing next-generation DNA sequencing data. *Genome research* **20**, 1297–1303 (2010).

16. A. Wilm, P. P. K. Aw, D. Bertrand, G. H. T. Yeo, S. H. Ong, C. H. Wong, C. C. Khor, R. Petric, M. L. Hibberd, N. Nagarajan, LoFreq: a sequence-quality aware, ultra-sensitive variant caller for uncovering cell-population heterogeneity from high-throughput sequencing datasets. *Nucleic acids research* **40**, 11189–11201 (2012).

17. S. Funke, A. Maisner, M. D. Mühlebach, U. Koehl, M. Grez, R. Cattaneo, K. Cichutek, C. J. Buchholz, Targeted cell entry of lentiviral vectors. *Molecular therapy : the journal of the American Society of Gene Therapy* **16**, 1427–1436 (2008).

18. B. Mrkic, J. Pavlovic, T. Rülicke, P. Volpe, C. J. Buchholz, D. Hourcade, J. P. Atkinson, A. Aguzzi, R. Cattaneo, Measles Virus Spread and Pathogenesis in Genetically Modified Mice (1998).

19. K. H. Dinnon, S. R. Leist, A. Schäfer, C. E. Edwards, D. R. Martinez, S. A. Montgomery, A. West, B. L. Yount, Y. J. Hou, L. E. Adams, K. L. Gully, A. J. Brown, E. Huang, M. D. Bryant, I. C. Choong, J. S. Glenn, L. E. Gralinski, T. P. Sheahan, R. S. Baric, A mouse-adapted model of SARS-CoV-2 to test COVID-19 countermeasures. *Nature*. 10.1038/s41586-020-2708-8 (2020).

20. A. B. Lyons, C. R. Parish, Determination of lymphocyte division by flow cytometry. *Journal of immunological methods* **171**, 131–137 (1994).

21. V. M. Corman, O. Landt, M. Kaiser, R. Molenkamp, A. Meijer, D. K. Chu, T. Bleicker, S. Brünink, J. Schneider, M. L. Schmidt, D. G. Mulders, B. L. Haagmans, B. van der Veer, S. van den Brink, L. Wijsman, G. Goderski, J.-L. Romette, J. Ellis, M. Zambon, M. Peiris, H. Goossens, C. Reusken, M. P. Koopmans, C. Drosten, Detection of 2019 novel coronavirus (2019-nCoV) by real-time RT-PCR. *Euro surveillance : bulletin Europeen sur les maladies transmissibles = European communicable disease bulletin* **25 (**2020).
